# Lignocellulosic Nanocrystals from Sawmill Waste as Biotemplates for Free-Surfactant Synthesis of Photocatalytically Active Porous Silica

**DOI:** 10.1021/acsami.2c02550

**Published:** 2022-04-20

**Authors:** Maryam El Hajam, Noureddine Idrissi Kandri, Abdelaziz Zerouale, Xiaoju Wang, Jan Gustafsson, Luyao Wang, Ermei Mäkilä, Leena Hupa, Chunlin Xu

**Affiliations:** † Processes, Materials and Environment Laboratory (PMEL), Faculty of Sciences and Techniques, Sidi Mohammed Ben Abdellah University, Road Imouzzer, BP 2202 Fez, Morocco; ‡ Signals, Systems and Components Laboratory (SSCL), Faculty of Sciences and Techniques, Sidi Mohammed Ben Abdellah University, Road Imouzzer, BP 2202 Fez, Morocco; § Laboratory of Natural Materials Technology, Åbo Akademi University, Henrikinkatu 2, FI-20500 Turku, Finland; ∥ Pharmaceutical Sciences Laboratory, Faculty of Science and Engineering, Åbo Akademi University, Tykistökatu 6A, FI-20520 Turku, Finland; ⊥ Laboratory of Industrial Physics, Department of Physics and Astronomy, University of Turku, FI-20520 Turku, Finland; # Laboratory of Molecular Science and Technology, Åbo Akademi University, Henrikinkatu 2, FI-20500 Turku, Finland

**Keywords:** sawdust, softwood, hardwood, cellulose nanocrystals, nanostructured porous silica, photocatalysis

## Abstract

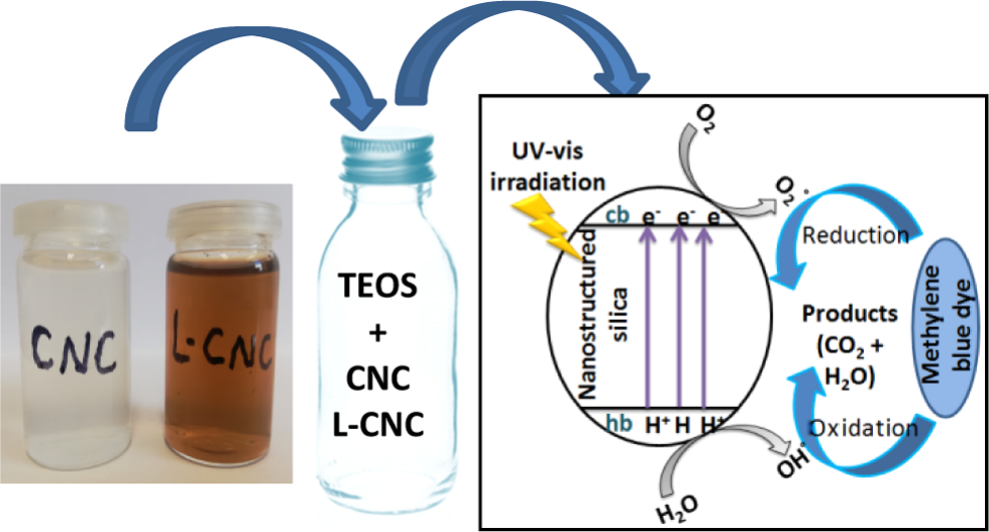

This work presents a new approach for more effective valorization of sawmill wastes (Beech and Cedar sawdusts), which were used as new sources for the extraction of lignin-containing and lignin-free cellulose II nanocrystals (L-CNCs and CNCs). It was shown that the properties of the extracted nanocrystals depend on the nature of the used sawdust (softwood or hardwood sawdusts). L-CNCs and CNCs derived from Beech fibers were long and thin and also had a higher crystallinity, compared with those obtained from Cedar fibers. Thanks to their interesting characteristics and their high crystallinity, these nanocrystals have been used without changing their surfaces as template cores for nanostructured hollow silica-free-surfactant synthesis for photocatalysis to degrade methylene blue (MB) dye. The synthesis was performed with a simple and efficient sol–gel method using tetraethyl orthosilicate as the silica precursor followed by calcination at 650 °C. The obtained materials were denoted as B/L-CNC/nanoSiO_2_, B/CNC/nanoSiO_2_, C/L-CNC/nanoSiO_2_, and C/CNC/nanoSiO_2_, when the used L-CNC and CNC cores are from Beech and Cedar, respectively. By comprehensive analysis, it was demonstrated that the nanostructured silica were quite uniform and had a similar morphology as the templates. Also, the pore sizes were closely related to the dimensions of L-CNC and CNC templates, with high specific surface areas. The photocatalytic degradation of MB dye was about 94, 98, 74, and 81% for B/L-CNC/nanoSiO_2_, B/CNC/nanoSiO_2_, C/L-CNC/nanoSiO_2_, and C/CNC/nanoSiO_2_, respectively. This study provides a simple route to extract L-CNCs and CNCs as organic templates to prepare nanostructured silica. The different silica structures showed excellent photodegradation of MB.

## 1 Introduction

Over the last two decades of progress in nanotechnology, significant efforts have been devoted to designing, monitoring, and controlling the synthesis of hierarchical core–shell nanostructured, mesoporous, and hollow materials.^1^ The hierarchical porous materials have attracted tremendous interest, owing to their outstanding properties, namely, their high specific surface areas and unique pore sizes. Accordingly, they are used in several applications, such as catalyst supports, fuel cells, drug delivery systems, luminescent and fluorescent materials, enzyme carriers, size exclusion chromatography, and adsorbents.^2^

There are various approaches to fabricating these materials. One of the most effective approaches is templated synthesis, which involves using nanoscale materials with defined shapes and sizes as reaction mediums.^3^ More precisely, the synthesis of core–shell materials is performed on the template’s surface. The hollow nanostructures will then be obtained by calcination or hydrolysis. A large variety of core templates have been applied to fabricate porous materials, for example, using metals,^4^ polymers,^5^ and inorganic, nonmetallic materials,^6^ based on the synthesis conditions and the final product properties. An ideal template material should have controlled and desired structure, shape, and size. In addition, it should maintain its shape in the synthesis medium and be simple to remove after the structure has been copied.^7^

Several types of natural materials such as bacteria,^8^ diatoms,^9^ chitin,^10^ living cells,^11^ cornstarch,^12^ pollen,^13^ and wood^14^ have been used as templates to mimic their specific shapes.

Sawdust residue of the timber processing industry (sawmills) is available in large quantities. Currently, it is mainly used as fuel in various energy fields or thrown recklessly into aquatic systems, thus generating a potential source of harmful air and aquatic pollution.^15^ Due to its abundant availability and low cost, sawdust finds potential as a raw material for several natural polymeric nanomaterials, such as cellulose nanocrystals (CNCs) and cellulose nanofibrils. CNCs can be isolated from different cellulose sources such as wood pulp, sawdust, sugar beet pulp, and cotton by various treatments, for example, acid hydrolysis.^16^

CNCs present various interesting physicochemical properties such as biodegradability, renewability, thermal stability, high-surface area, biocompatibility, their liquid crystalline character, and high mechanical properties and thus found to be promising in the fabrication of advanced materials with specific functionalities.^17^ For example, CNCs are often used as reinforcing materials in plastics^18^ and as biotemplates for preparation of hollow nanotubes and rodlike materials.^19^

Silica nanorod materials with their unique optical and electronic features are promising in high-tech industrial applications such as wave guides in microphotonic devices,^20^ nanoelectronics,^21^ bioseparation,^22^ and biosensing.^23^ Li et al.^24^ prepared the nanostructured silica materials using CNCs as a template via the sol–gel procedure for their applications in ultrahigh-molecular-weight polyethylene composites. Dujardin et al.^25^ successfully obtained mesoporous silica material after template removal by coating cellulose nanorod nematic suspensions with the SiO_2_ using sol–gel process. Zollfrank et al.^26^ also reported the synthesis of silica nanotubes using modified CNCs with oligopropylamino side chains in the medium of dimethyl sulfoxide as a template. Zhang et al.^27^ found that it was not easy to obtain mesoporous silica tubes based on untreated CNCs. In their study of mesoporous silica nanotube production, dual templates of native cellulose fibers, and cetyltrimethylammonium bromide (CTAB) micelles were used. The cellulose nanofibers were coated with an ultrathin layer of the titania film in order to improve the adhesion of CTAB on its surface and then to facilitate the sol–gel reaction of tetraethyl orthosilicate (TEOS) to prepare silica/CNC composites. All these studies are based on the use of modified lignin-free CNCs (L-CNCs) prepared from commercial microcrystalline cellulose I and CTAB surfactant as templates in the preparation of silica nanorod materials. The use of cellulose I nanocrystals requires the addition of a surfactant in order to modify their surface. In addition to the modification of the surface of cellulose I nanocrystals, the surfactant acts as a second template, which limits the actual value and role of the CNCs known by their original interesting properties. Hence, our objective complements the previous studies and searches/strives to use natural and more stable lignocellulosic nanocrystals (allomorph II) as promising biotemplates in free-surfactant synthesis of porous nanostructured silica for their applications as reusable photocatalysts for dye degradation.

In this paper, both lignin-containing and L-CNCs (CNCs with allomorph of cellulose II) were extracted from Cedar (softwood) and Beech (hardwood) sawdusts. Both types of CNCs were investigated as templates for surfactant-free synthesis of nanoporous silica materials with a simple, efficient, and low-cost sol–gel method where TEOS was used as a silica precursor of the SiO_2_ matrix. The morphology, geometric structure, pore size distribution, thermal stability, and specific surface area of the obtained nanoporous SiO_2_ materials were comprehensively studied. The prepared hollow silica nanorods were further tested for photocatalysis degradation of methylene blue (MB) dye.

## 2 Materials and Methods

### 2.1 Raw Materials

Locally available lignocellulosic wood sawdusts were acquired from a local sawmill industry in Fez city/Morocco on the same day of July 2019, directly after their sawing. These wood wastes belonging to Cedar (*Cedrus atlantica*) “softwood” and Beech (*Fagus sylvatica*) “hardwood” varieties were used in the extraction of L-CNCs and CNCs. Representative samples of each group were washed several times with water to remove the impurities from the surface, dried in sunlight for a couple of days, and then ground. The sawdust was sieved with a laboratory device to obtain different size ranges of particles. The fraction passing through 18 meshes (less than 1000 μm size screen) was selected for the first step of cellulose extraction (cooking). The pre-prepared samples were stored in a freezer at −24 °C prior to further use.

### 2.2 Reagents

Sodium hydroxide (NaOH) (99% purity) and sodium sulfide (Na_2_S) were used for alkaline treatment; sodium chlorite (NaClO_2_), glacial acetic acid, and sodium hydroxide (buffer solution) were used as bleaching agents, while sulfuric acid (95–98% purity) was used for acid hydrolysis. Dialysis membranes (cellulose membranes with an average flat width of 10 mm and molecular-weight cutoff: 6000–8000 Da) were used as received, tetraethoxysilane (TEOS, >95%) and ethanol (EtOH, >99.7%) were used during the silica preparation, MB dye (C_16_H_18_ClN_3_S, >95%) was used as a model pollutant in the photocatalysis study. Pure water from a Millipore Milli-*Q* Plus 185 purification system (resistivity 18.2 MU cm) was used for all experiments. All the chemicals purchased from VWR Chemicals and Sigma-Aldrich were of reagent grade and were used as received without any further purification.

### 2.3 Preparation of CNCs

The pre-prepared sawdust fibers from softwood and hardwood were cooked using an alkaline pulping approach in order to remove hemicellulose, lignin, and other impurities by saponification and cleavage of lignin–carbohydrate linkages. This delignification stage was accomplished using an alkaline leaching solution of NaOH/Na_2_S. Afterward, an additional bleaching step by sodium chlorite in acidic medium was conducted. Both the cooked and bleached pulps were subjected to a mechanical and acid hydrolysis process to obtain the colloidal suspensions of lignin-containing CNCs (L-CNCs) and lignin-free CNCs (CNCs), respectively. The denotations of the obtained L-CNCs and CNC samples based on different sources are C/L-CNC, C/CNC, B/L-CNC, and B/CNC.

### 2.4 Preparation of L-CNC and CNC-Silica Core–shell Composites

Both L-CNCs and CNCs were coated with conformal shells of amorphous silica in an acid-based sol–gel process using tetraethylorthosilicate (TEOS). TEOS (7.20 g) was added to glacial acetic acid (24.00 g) under stirring at room temperature for 2 min. The resulting mixture was added separately to an aqueous L-CNC and CNC dispersions (7.20 g, 0.6% w/w) and kept stirring for 60 min at a pH of 0.5–1.0. At the end of the reaction, the suspensions of the obtained silicated nanocrystals were washed and purified through repeated centrifugation with ethanol. The nanocomposites consisting of silica shells and L-CNCs or CNCs cores were obtained by air-drying the sediments at 50 °C under vacuum for 24 h. The obtained nanocomposites were denoted as C/L-CNC/SiO_2_, C/CNC/SiO_2_ (when the used L-CNC and CNC cores are from Cedar), B/L-CNC/SiO_2_ and B/CNC/SiO_2_ (when the used L-CNC and CNC cores are from Beech).

### 2.5 Fabrication of Nanostructured Porous SiO_2_ by Pyrolysis of Cellulosic Templates

The purified oven-dried L-CNC and CNC-silica core–shell materials were transformed to nanostructured porous SiO_2_ composites through calcination in air, at 650 °C for 6 h in a muffle furnace (Nabertherm, Germany) and cooled to room temperature at the rate of 2 °C/min to remove the organic compound parts (L-CNCs and CNC core templates). The organic moieties in the silica layer were released as CO_2_ and water vapor. The nanostructured hollow SiO_2_ obtained by calcinating C/L-CNC/SiO_2_ under air was denoted as (C/L-CNC/nanoSiO_2_). The other porous SiO_2_ were denoted as (C/CNC/nanoSiO_2_), (B/L-CNC/nanoSiO_2_), and (B/CNC/nanoSiO_2_).

### 2.6 Physicochemical Characterization

The surface morphology of raw, cooked, and bleached fibers obtained at the different steps of the chemical processes and the nanostructured porous silica were evaluated with scanning electron microscopy–energy-dispersive spectroscopy (SEM–EDS) (SEM, LEO Gemini 1530 SEM, Germany). The morphological properties (structure and size distribution) of L-CNCs and CNCs isolated from each cooked and bleached pulp-fiber, successively, and the nanostructured silica were examined with transmission electron microscopy (TEM, JEM-1400 Plus TEM, JEOL Ltd., Japan). The particle size distribution and the surface charge of the particles (zeta potential) of L-CNCs, CNCs, and nanostructured silica suspensions were measured using a Malvern Zeta sizer 3000 (United Kingdom).

The raw, cooked, bleached, and hydrolyzed samples and silica-coated L-CNCs and CNCs and nanostructured silica were analyzed with Fourier transform infrared (FTIR) and X-ray diffraction (XRD) using a Nicolet iS50 FTIR spectrometer (USA) equipped with Specac Golden Gate single-reflection attenuated total reflection accessory measurements and a Bruker Discover D8 X-ray diffractometer (Germany) using Cu Kα radiation (λ = 1.5406 Å), respectively.

The approaches used to determine the diffraction parameters of each sample were the same as those previously described, consisting of the empirical method proposed by Segal to determinate the crystallinity index CrI (eq 1)^28^ and the Scherrer equation (eq 2) to calculate the average thickness of cellulose crystallites (*D*_*hkl*_)^29^
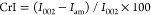
1

In this equation, CrI expresses the relative degree of crystallinity in (%), *I*_002_ is the maximum intensity of the crystalline peak at 2θ between 22 and 23 for cellulose I (between 18 and 22 for cellulose II), and *I*_am_ is the minimum intensity of the diffraction of the amorphous region at 2θ between 18 and 19 for cellulose I (between 13 and 15 for cellulose II).^30^
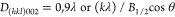
2where *D*_(*hkl*)_ is the crystallite size in nanometer perpendicular to the diffracting planes with Miller indices *hkl*, *K* is the constant of correction generally taken around 0.9, λ is the wavelength of X-ray radiation (λ = 0.15406 nm), β_1/2_ is the full-width half maximum of lattice plane reflection in radian, and θ is the corresponding Bragg angle (reflection angle).

Kappa number was measured according to Scan-C 1:00 (revised 2000).^31^ Weight percentages of elements (CHNS) in the different samples were determined using a Thermo Scientific Flash 2000 Organic Element analyzer.

A TA Instrument SDT Q600 Simultaneous Thermogravimetric analyzer was used to characterize the thermal stability [differential thermal analysis (DTA)/thermogravimetric analysis (TGA)] of the L-CNCs, CNCs, silica-coated L-CNCs or CNCs, and nanostructured silica samples.

Specific surface area, pore volume, and pore size distribution of nanostructured porous SiO_2_ were measured based on nitrogen sorption analysis at −196 °C, using Micromeritics 3Flex 3500 instrument.

### 2.7 Evaluation of the Photocatalytic Performance

Photocatalytic activity of the prepared nanostructured porous silica was evaluated by measuring the photodegradation of a cationic dye (MB), in a batch Pyrex reactor. Photocatalytic experiments were carried out at 25 °C by adding a given mass of the prepared materials to 100 mL MB solutions. Prior to the UV–vis irradiation step, the mixtures were vigorously stirred in the dark for 1 h to ensure the adsorption–desorption equilibrium between the nanostructured silica nanocomposite photocatalysts and dye solutions. After that, these suspensions were exposed to irradiation with UV–vis light for 240 min using a tungsten lamp 300 W, which was fitted on the top of the reactor and located at about 5 cm from the photo-catalysis system. The removal of MB in the dark and UV–vis irradiation was followed by collecting the aliquot solutions at a regular time interval of 20 min, and each solution was centrifuged and filtered through 0.45 μm glass fiber filters to remove the silica catalyst particles and then subjected to subsequent UV–vis spectrophotometric analysis at the wavelength of 664 nm to determine its corresponding absorbances in order to measure the dye degradation percentages.

The impact of several operational factors on the photodegradation efficiency such as the amount of catalysts at 0.25, 0.5, 0.75, and 1 g/L, the pH at 2, 4, 6, 8, and 10, the initial concentration of MB at 25, 50, 100, 150, and 200 mg/L, and time of light irradiation was studied.

The removal percentage of MB in the dark [RP (%)] and its degradation percentages in UV–vis [(DP) (%)] were achieved according to eq 3:
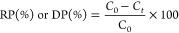
3where *C*_0_ and *C*_*t*_ are the initial and equilibrium concentrations of dye solutions, respectively.

The equilibrium concentration of MB after the removal in the dark was used as the initial concentration value for the photodegradation process.

## 3 Results and Discussion

### 3.1 Preparation and Characteristics of L-CNCs and CNCs

#### 3.1.1 Morphology, Particle Size, and Zeta Potential

**Figure 1:**
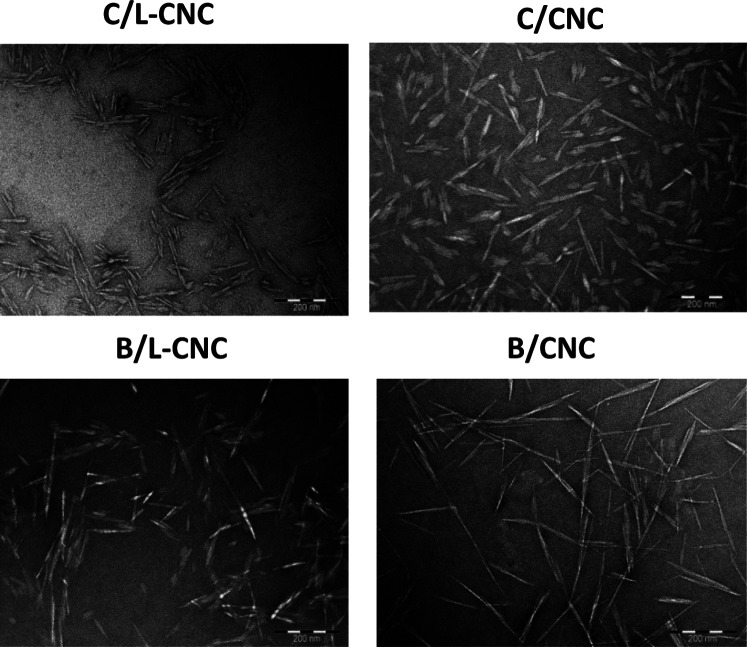
TEM of L-CNCs and CNCs.

TEM micrographs show clear nano-sized crystals for both Cedar and Beech sources and both types of CNCs in Figure 1. Well-separated nanocrystals are observed in the case of CNCs, owing to the increased surface charge density, critical for a good stability in the colloidal suspension.^32^ Some laterally aggregated elementary crystallites are observed in the case of L-CNCs, ascribed to aggregated lignin on the CNC surfaces acting as adhesive. L-CNCs and CNCs exhibited a typical non-uniform rodlike/needle-like shape, confirming that the extraction of nano-whiskers from wood sawdusts by acid hydrolysis was successful.

L-CNC and CNC from Cedar sawdust exhibit average lengths of 87 ± 18 and 96 ± 17 nm and diameters of 12 ± 2 and 9 ± 2 nm, respectively, resulting in aspect ratios of 7.25 for L-CNC and 10.66 for CNC. In contrast, the lengths and diameters of nanocrystals in the case of Beech sawdust were about 145 ± 22 and 9 ± 2 nm for L-CNC and about 155 ± 25 and 4.8 ± 0.9 nm for CNC, respectively, yielding aspect ratios of about 16.11 for L-CNC and 32.29 for CNC (Figure S6).

The size and shape of nanocrystals depend strongly on the fiber source. The nanocrystals extracted from Cedar sawdust were displayed in irregular rodlike shape rather than needle-like shapes obtained in the case of Beech sawdust.

The particle size distribution observed by dynamic light scattering (Figure S7) is consistent with the finding in TEM analysis. In the case of Cedar, L-CNC, and CNC, medium lengths of 81 and 92 nm were observed, respectively. However, lengths of 120 and 150 nm were exhibited for L-CNC and CNC, respectively, in the case of Beech. The presence of small peaks around 4000 and 1000 nm in the case of CNC extracted from Cedar could be related to some micron-scale particles in the suspensions.^33^ However, these peaks disappeared in the graphs, representing the volume distributions, which signify the absence of micron-sized particles, which might have resulted from the agglomeration of C/CNC during the size analysis.

Zeta potential (ζ-potential), a good indicator of the hydrolysis efficiency, was used to assess the surface charge of CNCs that is critical for the colloidal stability of the nanoparticle suspensions in the aqueous phase. The ζ-potential was about −18.9 and −39.6 mV for L-CNC and CNC extracted from Cedar and about −21.7 and −45.9 mV for Beech L-CNC and CNC, respectively (Table 1). During the treatment of the cellulosic and lignocellulosic pulps by H_2_SO_4_, the hydrolyzation of the amorphous parts of the cellulose causes the partial substitution of the surface hydroxyl groups with sulfate groups and results in a negative charge of the nanocrystal surface.^34^ Thus, the electrostatic repulsion force between the two electrical layers prevents nanocrystals from agglomeration and ensures good dispersion of these layers in the aqueous media and most of the polar solvents, such as DMF.^16^ CNCs exhibited a significantly lower ζ-potential than L-CNCs in both sources, which suggests that the residual lignin could have partially prevented the sulfonation of the cellulosic structure due to surface coverage. This plainly emphasizes that the colloidal dispersions of the CNCs were considered electrically stable because the absolute values of their zeta potentials exceeded the 25 mV.^35^ In contrast, it may be inferred that the L-CNC suspensions are less stable since they exhibited absolute values of zeta potentials less than 25 mV because of the fewer anionic sulfate groups on their surface.

**Table 1:** Crystallinity Index, Crystallites Sizes, Zeta Potential, Lignin Content, and Elemental Analysis

	Cedar					Beech				
sawdust	raw	cooked	bleached	L-CNC	CNC	raw	cooked	bleached	L-CNC	CNC
CrI (%)	41.24	71.49	73.99	62.07	65.86	40.83	71.37	76.14	75.59	79.47
*D*_002_ (nm)	3.09	3.76	3.95	2.32	3.25	2.94	4.19	5.40	5.68	5.91
zeta potential (mV)				–18.9	–39.6				–21.7	–45.9
Kappa number	237	41.8	2.4	35.9	1.4	161.5	27.5	1.5	23.5	1
lignin (%)	35.53	6.26	0.3	5.38	0.21	24.21	4.12	0.225	3.52	0.15
nitrogen (% w/w)	0.29	0.05	0.02	0.07	0.07	0.23	0.02	0.02	0.07	0.07
carbon (% w/w)	49.3	41.7	41.6	42.8	42.4	45.0	41.2	41.3	41.9	41.9
hydrogen (% w/w)	6.1	6.0	6.2	6.1	5.9	5.7	6.1	6.2	6.1	6.1
sulphur (% w/w)	0.00	0.00	0.00	0.55	0.67	0.00	0.00	0.00	0.64	0.82

XRD analysis shows that L-CNCs and CNCs from both sources are in the form of cellulose II, which is characterized by short lengths in comparison with cellulose I, and it can be shown in different shapes, rodlike, needle-like, elliptical-like, or granule-like.^36,37^ The short lengths of this kind of cellulose were mainly caused by their special crystalline structure,^38^ induced by the deeper degree of the acid hydrolysis, which might induce a decrease in the DP.

#### 3.1.2 Structural Characteristics

**Figure 2:**
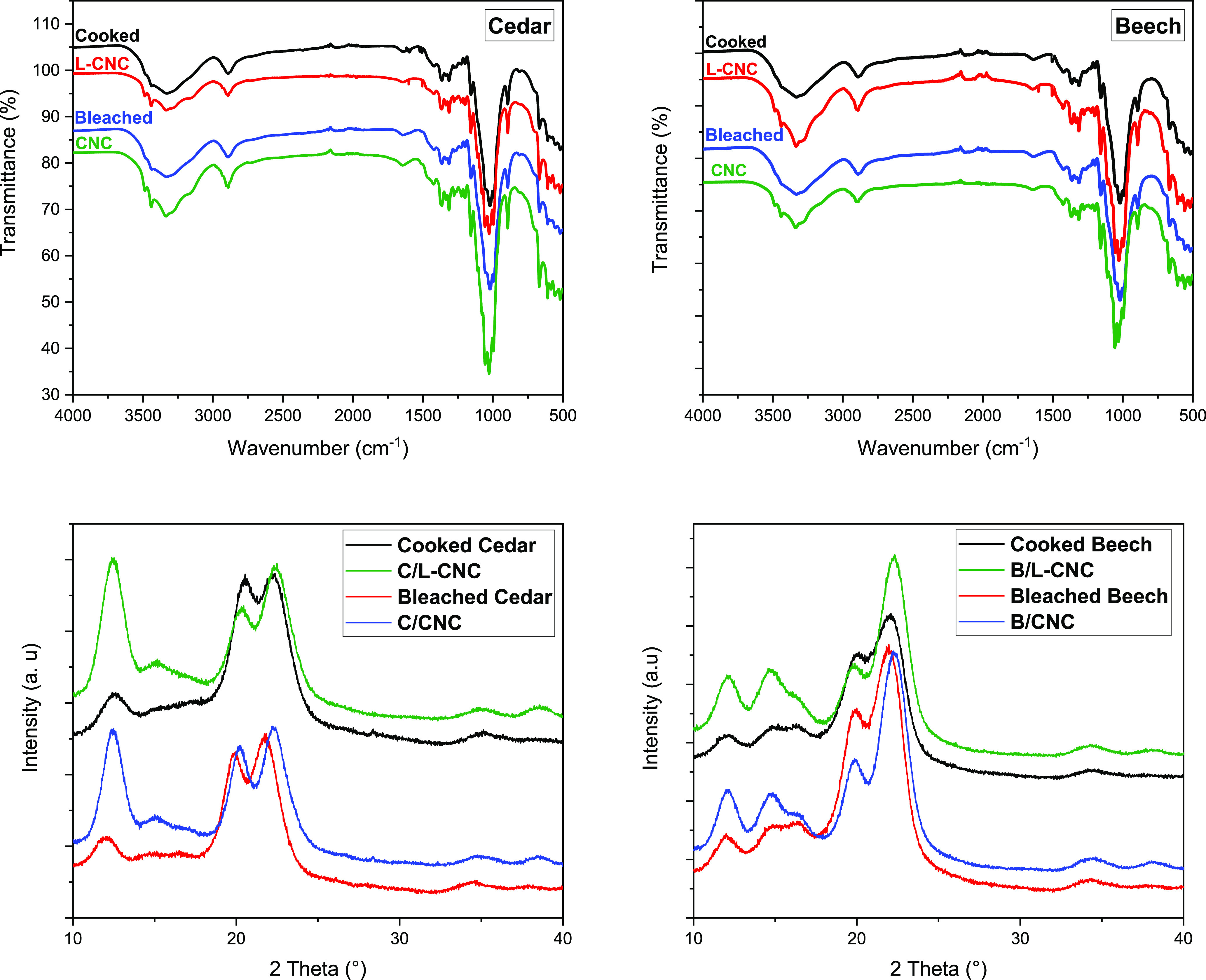
FTIR spectra and XRD diffractograms of L-CNCs and CNCs extracted from Cedar and Beech sawdusts.

FTIR spectra of L-CNCs and CNCs of both sources exhibited almost the same sharp features of cellulose macromolecules with some differences in the band intensities and the appearance of new slight absorption peaks, suggesting that acid hydrolysis induces slight chemical changes in L-CNCs and CNCs (Figure 2).

Beech L-CNC and CNC exhibit a narrower and stronger peak in the region related to OH vibrations around 3400 cm^–1^, due to the increase in the hydrogen bond strength caused by the removal of the amorphous components and the subsequent increase in crystallinity.^39^ However, in the case of Cedar L-CNC and CNC, these intensities gradually decreased, indicating that the hydrolysis process not only removed the amorphous portion of cellulose but probably disrupted the crystalline parts and the hydrogen bonds. The hydrolysis likely decreased the CrI and crystallite sizes and hence decreased the peak intensities.^40^

The chemical alterations were indicated by the change in FTIR spectra for free hydroxyl groups in the range of between 3100 and 3400 cm^–1^. Compared with cooked and bleached pulps, the spectra of isolated L-CNCs and CNCs presented small bands at 3486 and 3436 cm^–1^ that could be assigned to the intramolecular hydrogen bonding in between C3OH–OC6 (minor component) and C3OH–OC5 (major component), respectively, in cellulose II.^41^ On the other hand, the peaks appeared at 3280 cm^–1^ in the case of L-CNC and CNC extracted from Beech and at 3154 cm^–1^ in the case of those extracted from Cedar, signifying the new intermolecular bonding pattern between C2OH–OC6 and C6OH–OC2, correspondingly.^41,42^ Another difference that can be detected in L-CNCs and CNCs spectra compared to those of cooked and bleached pulps, successively, is the appearance of a new small absorption band at 1206 cm^–1^, which is associated with the S=O stretching from residual sulfate groups. This confirms the occurrence of the esterification reaction of the hydroxyl groups of cellulose during the hydrolysis process; thus, the presence of sulfonate groups at the L-CNCs and CNCs surface is confirmed. In addition, the observation of peaks at 996 cm^–1^ in the spectra of all L-CNCs and CNCs was due to the reduction in amorphous zones in the polysaccharide matrix by acid hydrolysis.^43^ This peak stands for cellulose II and can be assigned to β-glycosidic linkages between glucose units in cellulose. Compared to the isolated cellulose, it can be concluded that the structure of cellulose II was significantly ameliorated in L-CNCs and CNCs. The residual lignin in L-CNCs has been observed by the small characteristic bands around 1515 and 1602 cm^–1^, which were absent in the L-CNCs. The changes in the crystalline structure and related crystallinity indexes of L-CNCs and CNCs were investigated by XRD analysis (Table 1).

XRD patterns of L-CNCs and CNCs were similar to those of cooked and bleached pulps, respectively; however, differences in their relative intensities were notable. The diffractograms of both Cedar L-CNCs and CNCs exhibit three major diffraction peaks at around 12.3, 20.4, and 22.5°, corresponding to the cellulose II crystallographic planes (1–10), (110), and (020), successively. However, the diffraction profiles of L-CNC and CNC in the case of Beech contained characteristic peaks of both cellulose I (2θ = 14.9 and 16.3°) and cellulose II (2θ = 12.5, 20.5, and 22.7°).^44^ These results indicate that L-CNC and CNC extracted from Cedar were mainly cellulose II, but those extracted from Beech pulps were mixtures of cellulose I and II, which is similar to the crystalline structure of their original cooked and bleached pulps. As shown in Table 1, the CrI of Cedar L-CNC and CNC decreased from 71.49 to 62.07 and 73.99 to 65.86%, respectively, in comparison with their cooked and bleached pulps. However, the CrI values registered a slight increase in the case of Beech L-CNC and CNC, reaching 75.59 and 79.47%, successively. It was also shown that the crystallinities of L-CNCs were lower compared to those of CNCs for both sources, which could be explained by the amorphous nature of lignin in the L-CNCs. The crystallites size (*D*_002_) of Beech L-CNC and CNC increased; however, they knew a slight decrease in the case of Cedar L-CNCs and CNCs.

A slight increase in CrI and *D*_002_ for Beech samples after acid treatment was mainly due to the removal of amorphous domains of cellulose. However, in the case of L-CNC and CNC from Cedar, the decrease in CrI and *D*_002_ indicates that the hydrolysis conditions employed removed the amorphous portion of cellulose and partially disrupted the crystalline parts.

Moreover, Kappa numbers and elemental analysis were conducted (Table 1). The high content of lignin was preserved in L-CNCs of both Cedar and Beech and was determined to be 35.53 and 24.21%, respectively. In contrast, lignins in CNCs from bleached pulps were nearly completely removed. As exhibited by CHNS element analysis, the carbon and nitrogen contents of L-CNCs and CNCs did not change significantly after the acid hydrolysis. Sulfur was registered in both L-CNCs and CNCs of both Cedar and Beech, and the content for CNCs was higher than that for L-CNCs in both sources. These results are in agreement with the zeta potential. The presence of the sulfate groups in nanocrystals was also confirmed by the peak in FTIR spectra at 1206 cm^–1^.

#### 3.1.3 Thermal Characteristics

**Figure 3:**
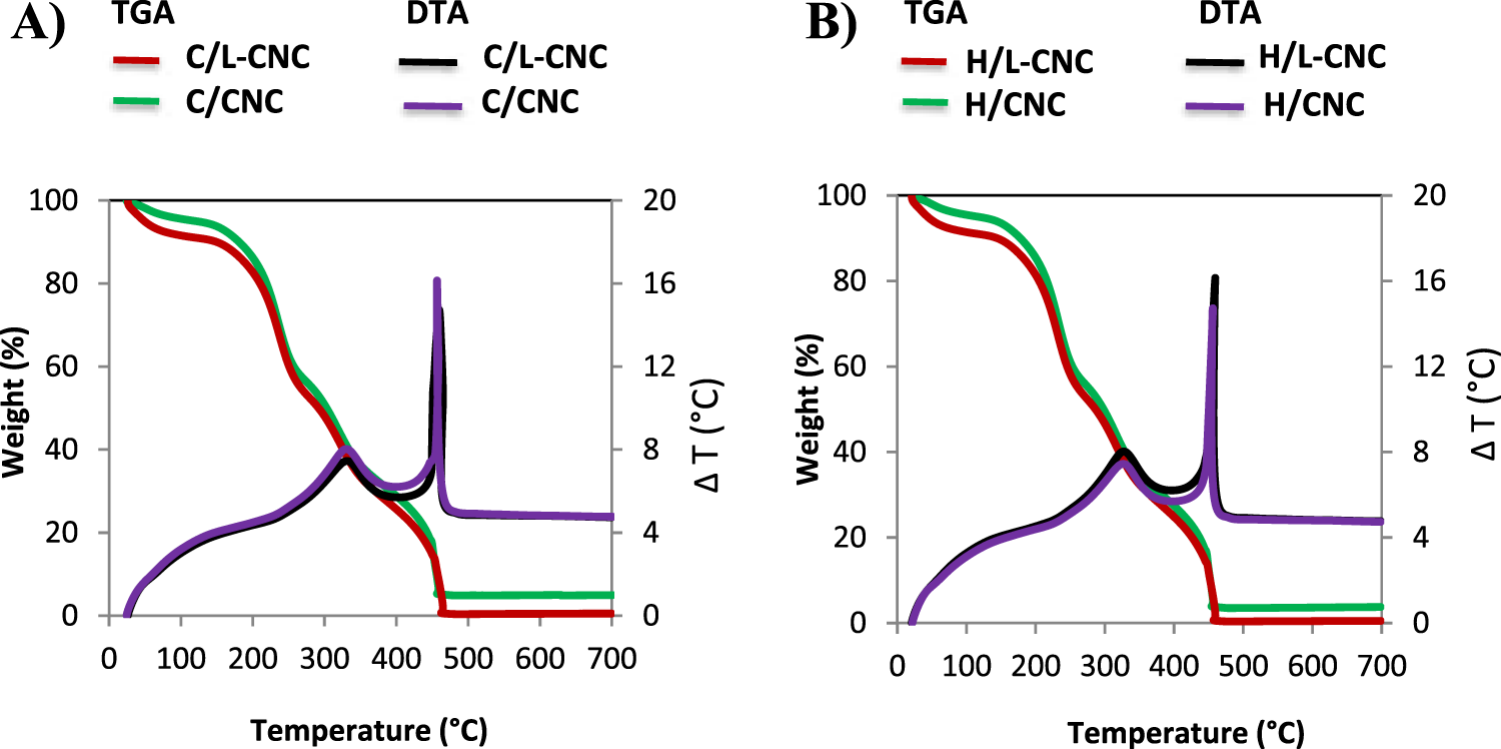
TG and DTA curves of L-CNCs and CNCs extracted from Cedar (A) and Beech (B).

The TGA thermograms of L-CNCs and CNCs extracted from both Cedar and Beech (Figure 3) showed less than 5 and 10% of weight loss in the range of 30–150 °C, respectively. The weight loss corresponds to the evaporation of the residual absorbed moisture, including chemisorbed water and/or the intermolecularly H-bonded water.^45^ The thermal behavior of L-CNCs and CNCs was approximately similar to that described in the literature using the same method of extraction from pulp fibers.^46^ All L-CNCs and CNCs exhibited gradual thermal transitions in the temperature region of 200–380 °C, principally assigned to the pyrolysis of cellulose itself.^47^ During cellulosic chain degradation, several steps take place, such as depolymerization, dehydration, and decomposition of glycosidic units.^48^ The first degradation process between 200 and 240 °C is assigned to the sulfate groups that catalyze the dehydration process of cellulose, and the second one found around 240–350 °C is related to the decomposition of the more accessible region inside unsulfated crystalline domains. In addition, the weight loss at temperature range from 350 to 500 °C is attributed to the breakdown of glycosidic bonds and carbon-containing skeleton with formation of charred residue.^49^

The relatively high amount of residues in CNCs in comparison with L-CNCs might be explained by the presence of sulfate groups in CNCs more than that in L-CNCs, as suggested by the CHNS results. These act as flame retardants in cellulose combustion and make char formation easy by promoting dehydration reactions.^50^ DTA curves of L-CNCs and CNCs also revealed the presence of two major weight loss stages between 250–400 and 400–500 °C, respectively. The first pyrolysis stage was mainly attributed to the degradation of cellulose chains, and the second one was caused by the slow carbonization of cellulose.^51^ The thermal degradation processes of L-CNC and CNCs from Cedar and Beech were similar.

### 3.2 Preparation and Characterization of Silica-Coated L-CNCs and CNCs and Hollow Silica

#### 3.2.1 Morphology

L-CNCs and CNCs were applied as templates for the preparation of nanostructured porous silica by a coating process. The synthesis procedure was carried out through an acid-based sol–gel method using TEOS as a molecular silica precursor. During the coating experiments, slight turbidity appeared a few minutes after adding L-CNCs and CNCs, proving the deposition of silica shells on L-CNC or CNC cores. However, no silica was obtained after centrifugation in the absence of cellulosic nanocrystals from the reactive medium. The solutions formed a gel within 2 days, which is in agreement with a standard sol–gel process.^52^ It can be explained that the presence of L-CNC or CNC templates led to a fast hydrolytic conversion of TEOS into core–shell L-CNC/SiO_2_ and CNC/SiO_2_ composites under acidic conditions. The same processes were executed on all L-CNC and CNC templates for comparison.

**Figure 4:**
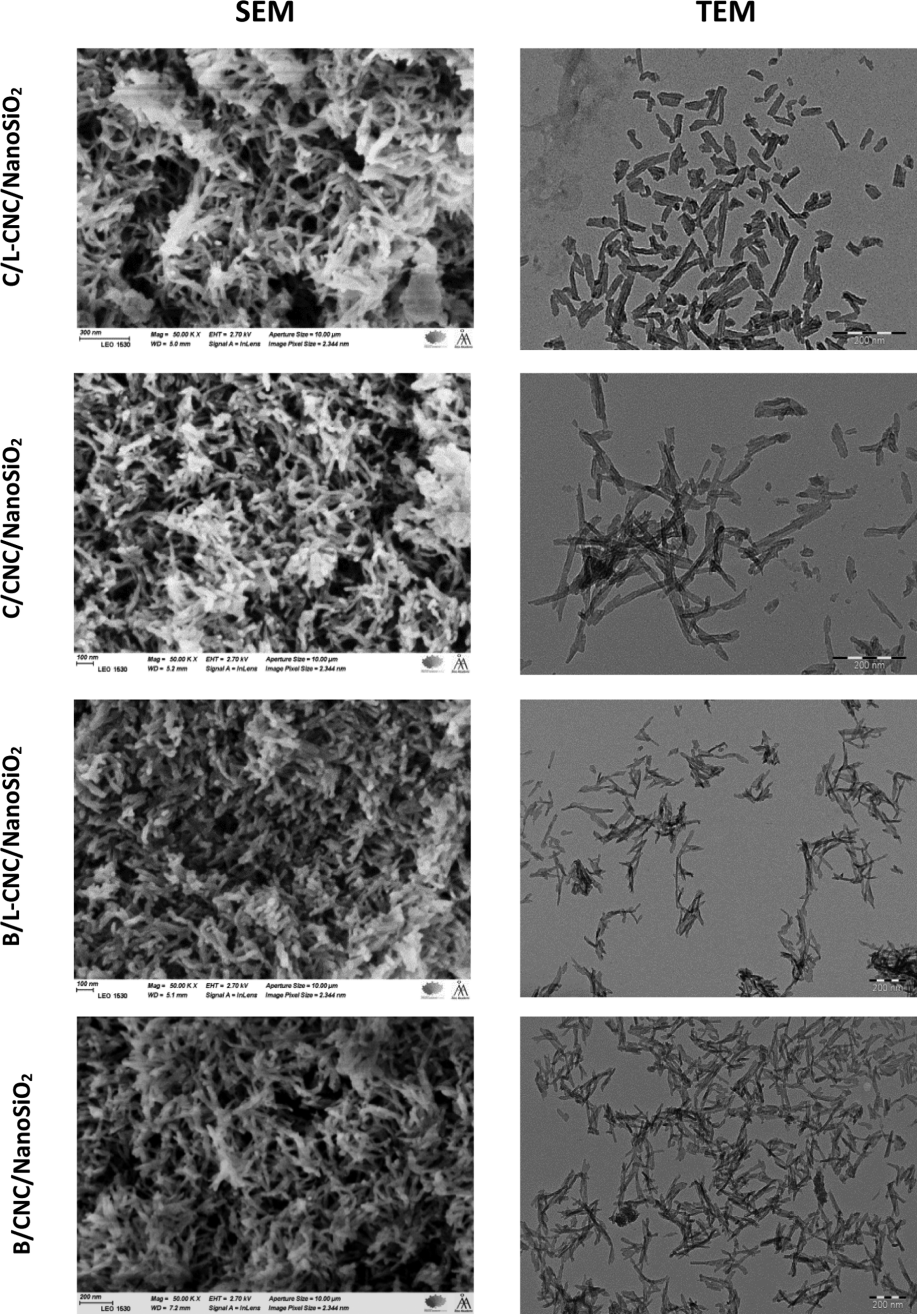
SEM and TEM micrographs of the different silica nanocomposites.

The SEM and TEM micrographs (Figure 4) show that individual L-CNCs and CNCs were successfully coated with silica shells, and their morphologies and sizes appeared different. The nanostructured porous silica was rodlike when both L-CNC and CNC templates were from Cedar. In contrast, needle-like structures were observed when Beech L-CNC and CNC were used as the template. This is ascribed to the preserved template structures that determine the core structure of porous silica materials. EDS (analysis) proved that the coating consisted of Si and O (Figure S8).

Particle size distribution observed by DLS analysis suggests that the lengths of nanostructured silica are in the range from 80 to 100 nm when the used templates are prepared from Cedar and from 150 to 200 nm when those are prepared from the Beech source (Figure S9).

Based on TEM results and ImageJ analysis, the average inner diameters of hollow nano-silica were about 12, 9, 9, and 5 nm, which are close to the diameters of L-CNCs and CNCs prepared from Cedar and Beech, successively (Figure S10). These results confirm the success of the use of L-CNCs and CNCs as templates.

#### 3.2.2 Structural Characteristics

**Figure 5:**
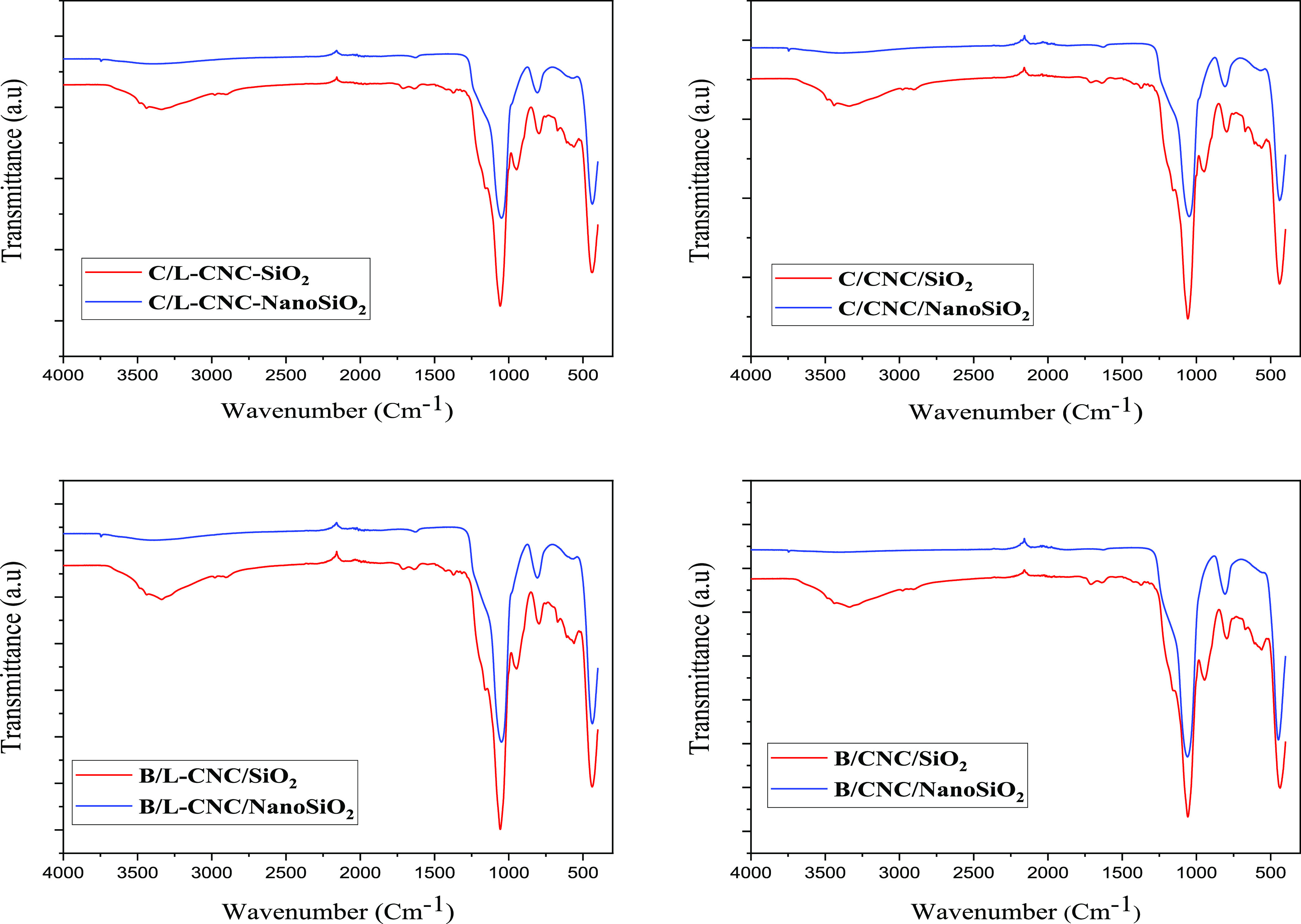
FTIR spectra of silica-coated L-CNCs and CNCs before and after calcination.

The FTIR spectra of silica-coated L-CNCs or CNCs and nanostructured porous silica are illustrated in Figure 5.

The spectra of nanostructured porous silica exhibited characteristic absorbance bands of SiO_2_ vibrational modes at around 436, 800, and 1048 cm^–1^, corresponded to the rocking vibration, symmetric stretching vibration, and asymmetric stretching vibration, respectively, of the Si–O–Si bond according to silica peaks reported in the literature.^53^ Characteristic cellulose bands were detected in silica-coated L-CNC and CNC patterns, such as C–H and CH_2_ symmetric and asymmetric stretching vibrational bands were observed at 2900 and 2969 cm^–1^. These bands completely disappeared in the calcined silica nanocomposites, indicating that the organic cellulose cores were removed by calcination. The adsorption band at 963 cm^–1^, corresponding to the bending vibration of the Si–OH bond, disappeared also completely after calcination. Moreover, the total disappearance of the O–H stretching bond around 3400 cm^–1^ is related to the loss of adsorbed water. As the Si–O–Si bonds mainly form during the hydrolysis and condensation process of TEOS with CNCs and L-CNCs as templates, during the high-temperature calcination, mainly the elimination of chemical bonds of −C–O–, −C–H–, and H–O–H through the combustion process was observed. The calcination process led to the total removal of the L-CNC and CNC templates and water, yielding nanostructured porous silica. After zooming on the spectra, we observe that the −Si–O–Si–O bands of nanostructured porous silica are a bit large in comparison with those of the silica coating, majorly because we have higher concentration of amorphous SiO_2_ in the samples when they are calcined.

**Figure 6:**
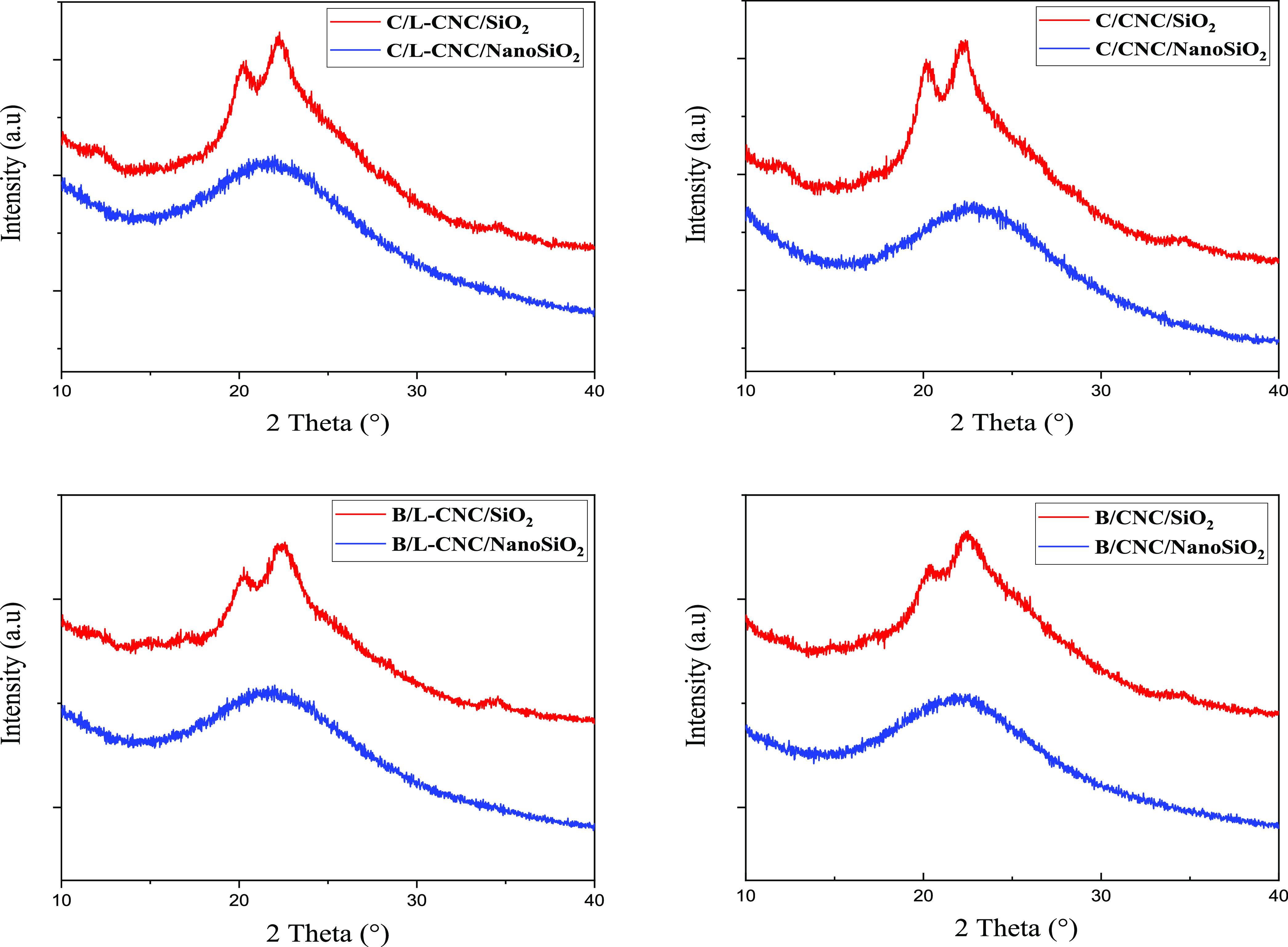
X-ray diffractograms of silica-coated L-CNCs and CNCs before and after calcination.

The removal of L-CNCs and CNCs was also confirmed through a comparison of the X-ray diffractograms of silica-coated L-CNCs or CNCs before and after calcination at 650 °C (Figure 6). Before calcination, the XRD patterns of core–shell L-CNC or CNC-silica (L-CNC/SiO_2_ and CNC/SiO_2_ composites) have smaller peaks at 20.2 and 22.5°, corresponding to the characteristic peaks of L-CNC or CNC templates combined with a wide peak of silica.^54^ The crystallinities of the core–shell L-CNC or CNC-silica were reduced due to the presence of amorphous silica. After calcination at 650 °C, only the broad halo centered at 22.2° remains in the diffractogram of hollow silica nanocomposites, which is typical for amorphous silica, as reported in the literature.^55^ Moreover, there were no new diffraction peaks, confirming that the SiO_2_ shell of the composite was amorphous.

#### 3.2.3 Nitrogen Sorption

Nitrogen sorption isotherm shows the mesoporous characteristics of the nanostructured silica with the hysteresis loops within relative pressure range 0.4 < *p*/*p*° < 1 for all materials (Figure S11).

The specific surface area of the prepared nanostructured porous silica using Cedar L-CNC and CNC templates was smaller than that of porous silica using Beech templates. The specific surface areas were 348 ± 3 and 392 ± 9 m^2^/g for C/L-CNC/nanoSiO_2_ and C/CNC/nanoSiO_2_, respectively, whereas those of B/L-CNC/nanoSiO_2_ and B/CNC/nanoSiO_2_ were about 953 ± 20 and 1107 ± 24 m^2^/g, successively. Nanostructured silica prepared using Cedar L-CNC and CNC possesses larger pore volumes than those obtained using Beech L-CNC and CNC due to the larger template sizes. The pore volumes of C/L-CNC/nanoSiO_2_ and C/CNC/nanoSiO_2_ were 1.98 and 1.71 cm^3^/g, respectively, while those of B/L-CNC/nanoSiO_2_ and B/CNC/nanoSiO_2_ were 1.17 and 1.15 cm^3^/g, respectively (Table 2). The high-surface areas of these porous silica nanomaterials are promising for applications such as photocatalytic dye degradation.

**Table 2:** Specific Surface Area and Total Pore Volume of Porous SiO_2_ Materials

sample	specific surface area (m^2^/g)	pore volume (cm^3^/g)
C/L-CNC/nanoSiO_2_	348 ± 3	1.98 ± 0.3
C/CNC/nanoSiO_2_	392 ± 9	1.71 ± 0.03
B/L-CNC/nanoSiO_2_	953 ± 20	1.17 ± 0.12
B/CNC/NanoSiO_2_	1107 ± 24	1.15 ± 0.07

The Brunauer–Emmett–Teller (BET) analysis shows a huge difference among the specific surface areas of C/L-CNC/nanoSiO_2_, C/CNC/nanoSiO_2_, B/L-CNC/nanoSiO_2_, and B/CNC/nanoSiO_2_ even if their preparation process is same. Because the only difference in the preparation process is the source of the template, we have attributed the difference between the resulted nanostructured silica to the physicochemical characteristics of L-CNCs and CNCs extracted from Cedar (softwood) and Beech (hardwood).

#### 3.2.4 Thermal Stability

**Figure 7:**
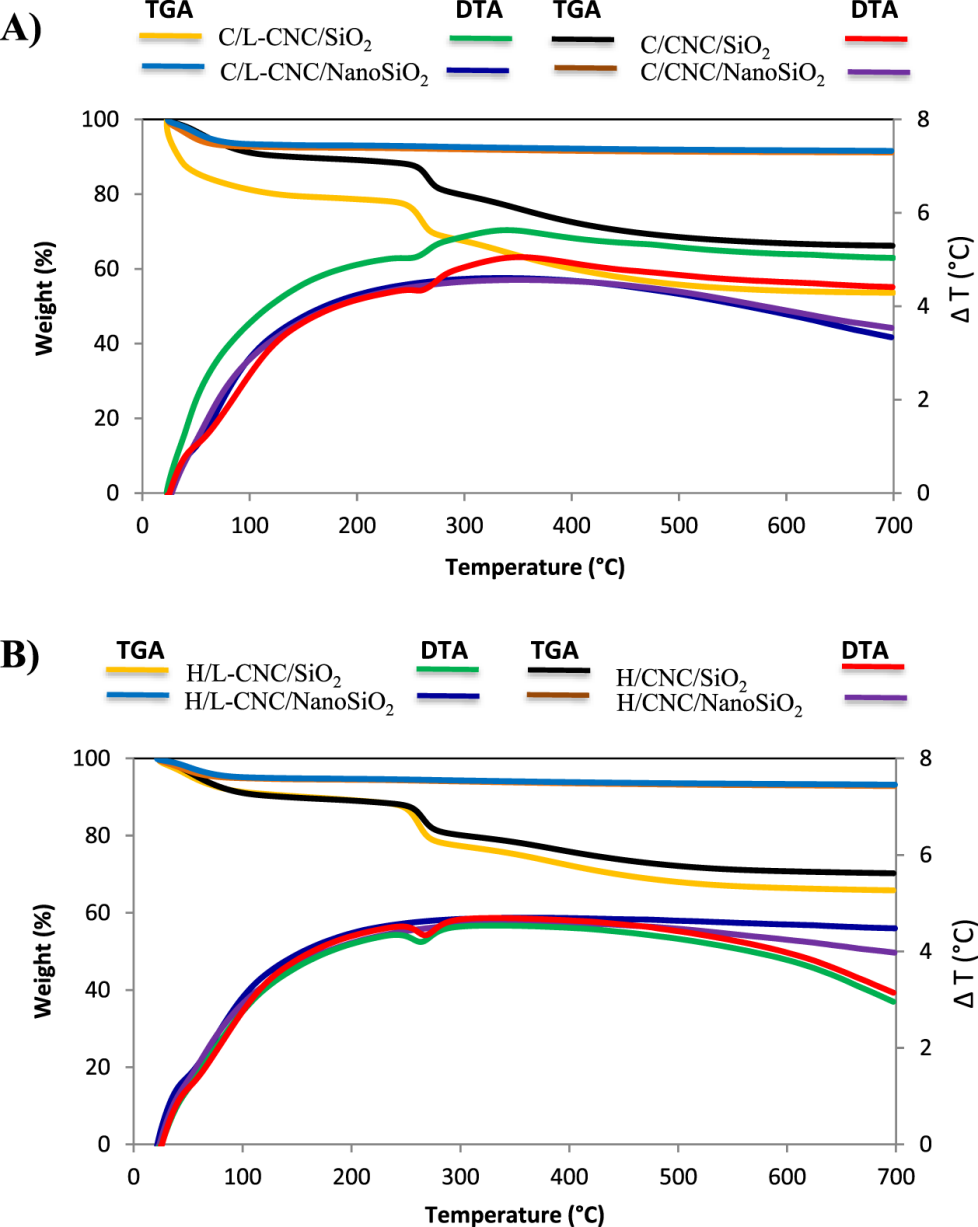
TG and DTA curves of silica-coated L-CNCs and CNCs extracted from Cedar (A) and Beech (B) and hollow silica nanocomposites.

After coating with silica, the thermal stability of L-CNCs and CNCs was significantly improved. The weight loss observed below 100 °C corresponds to the loss of adsorbed water. The presence of physisorbed water is very important, and reactivity of silane with silica is strongly influenced by physisorbed water.^56^ The pyrolysis of silica-coated L-CNCs and CNCs exhibited two different stages. The first stage, 250–350 °C, is attributed to the decomposition of L-CNCs and CNCs. The second stage, between 350 and 500 °C, corresponds to the slow carbonization process of L-CNCs and CNCs and degradation outcomes. The weight remained constant after 500 °C. The curves of nanostructured porous silica are the most stable ones among the TGA and DTA curves, showing almost horizontal lines (Figure 7).

### 3.3 Evaluation of the Photocatalytic Performance

In order to optimize the photocatalytic power of the different synthesized nanostructured silica, the impact of several operational key factors such as the amount of the catalysts, the initial dye concentration, pH of solution, and time of light irradiation were studied to achieve the maximum degradation percentage of MB.

#### 3.3.1 Study of the Concentration Effect

**Figure 8:**
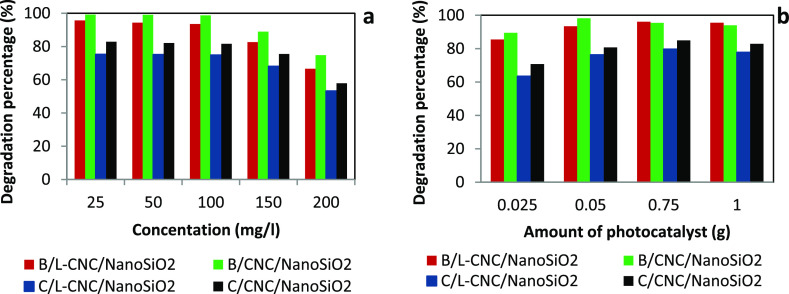
Effect of the initial concentration of MB (a) and amount of the catalyst (b) on the degradation percentage of MB.

The effect of the initial dye concentration on the MB degradation efficiency was studied in the MB concentration range of 25–200 mg/L for solution containing 0.05 g of each photocatalyst separately at a pH of 8 for 60 min in the dark and exposed to UV–vis light for 240 min. Then, the percentage of degradation under UV–vis light illumination was measured (Figure 8a). It appeared that the degradation percentages of MB reached their maximum of about 98, 94, 82, and 75% for B/CNC/nanoSiO_2_, B/L-CNC/nanoSiO_2_, C/CNC/nanoSiO_2_, and C/L-CNC/nanoSiO_2_, respectively. The degradation remained almost constant with the increase in the dye concentration up to 100 mg/L, which could be explained by the number of active species and enough electron–hole pairs. However, any further increase in the MB concentration above this limit caused the solutions to become opaque, and the degradation percentage of MB started to decrease because the energy resulting from light became insufficient to allow photocatalyst binding to the whole dye molecules. The same conclusion has been reported by Soltani-nezhad et al.^57^ Thus, the optimum MB concentration of 100 mg/L was chosen to be used in subsequent experiments.

#### 3.3.2 Study of the Catalyst Amount

A range of catalyst doses between 0.025 and 0.1 g with a step of 0.025 g per 100 mL of dye at 100 mg/L and pH of 8 was tested to investigate the effect of nanostructured silica catalyst concentration on the photocatalytic degradation of MB. As shown in Figure 8b, the MB degradation percentage increased with the catalyst amount, up to 0.05 g for B/CNC/nanoSiO_2_ with a degradation percentage of about 98%. Correspondingly, 0.075 g of B/L-CNC/nanoSiO_2_, C/CNC/nanoSiO_2_, and C/L-CNC/nanoSiO_2_ gave MB degradation percentages of about 93, 80, and 76%, respectively. Any further increase in the amount of catalysts induced a decrease in the degradation efficiency, which is consistent with the previously reported studies.^58,59^ As the amount of the catalyst increased, the number of the catalyst surface-active sites available increased. Therefore, the pored volume and the concentration of hydroxyl and superoxide radical increased, which led to high MB degradation percentage.^60^ However, when the mass exceeded the maximum value, the transparency of the solution decreased because of the increase in turbidity. This prevented the light penetration into the dye and the activation of the whole catalyst suspension.^59^ Accordingly, the MB degradation efficiency decreased.

In the case of B/L-CNC/nanoSiO_2_, C/CNC/nanoSiO_2_, and C/L-CNC/nanoSiO_2_ catalysts, 0.075 g did not greatly affect the degradation percentage in comparison with a lower amount of 0.05 g; thus, the catalyst amount of 0.05 g was chosen for further studies.

#### 3.3.3 Study of the pH Effect

**Figure 9:**
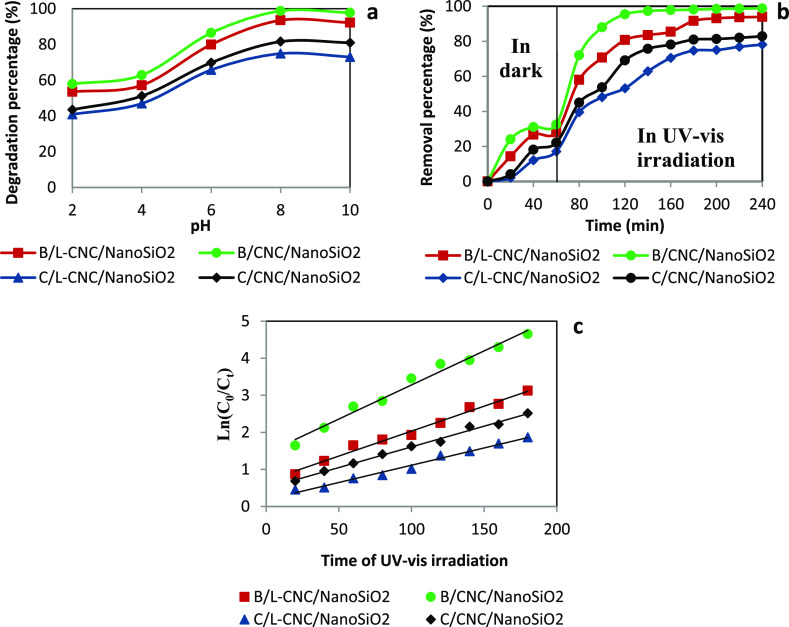
Effect of pH (c) and contact time (d) on the degradation percentage of MB, as well as the kinetics of MB degradation (e).

The effect of initial MB solution pH on its degradation was investigated by varying the pH of the MB solutions from 2 to 10 by adding 1 N HNO_3_ and 1 N NaOH using 100 mL of MB solution at 100 mg/L and 0.05 g of the catalyst. The mixtures were kept under agitation in the dark for 60 min, and then, they were exposed to UV–vis light illumination for 240 min. The degradation percentage was measured (Figure 9a). The degradation percentage of MB increased significantly up to pH 8, which corresponded to the maximum degradation of MB. After that, the degradation rate remained constant at 98, 94, 80, and 74% for B/CNC/nanoSiO_2_, B/L-CNC/nanoSiO_2_, C/CNC/nanoSiO_2_, and C/L-CNC/nanoSiO_2_, respectively. MB is a cationic dye and becomes positively charged when dissolves in water. However, the surface of the prepared nanostructured silica is negatively charged at pH higher than the zero-point charge (pHpzc), which is about almost 4 for the different synthesized materials. At a pH lower than 4, the surface of nanostructured silica catalysts is positively charged, whereas at a pH higher than 4, it becomes negatively charged. Therefore, a pH higher than that corresponding to the zero-point charge favors the adsorption of MB molecule on the catalyst surface, indicating improved degradation under basic conditions. In addition, with higher pH, the degradation of MB is also increased due to the deportation of the hydroxyl species present in the solution and their interaction with the dye molecules, resulting in the maximum degradation of MB.^61^ However, a lower degradation rate at a pH below 8 can be explained by the reduction of the hydroxyl groups in the solution, which was also reported previously by Konstantinou and Albanis.^62^

#### 3.3.4 Study of the Time Effect

Figure 9b shows the removal percentage of MB as a function of time before and when exposing the mixture of MB solution and the catalyst material to UV–vis irradiation. The other parameters were fixed in their optimum values: initial concentration 100 mg/L, pH 8, and catalyst amount 0.05 g. UV–vis irradiation was turned on after 60 min, which represent the removal time in the dark (adsorption stage).

As clearly shown, the MB removal percentage increased with the degradation time and then reached a saturation value. When compared with the adsorption rate under darkness, the MB degradation percentage under UV–vis illumination was much higher, proportionating to their adsorption rate. The same conclusion was reported previously.^63,64^

The removal rates under the dark condition over B/CNC/nanoSiO_2_, B/L-CNC/nanoSiO_2_, C/CNC/nanoSiO_2_, and C/L-CNC/nanoSiO_2_ catalysts were low and reached 32, 28, 22, and 17%, respectively, in 40 min and then remained constant, proving that the adsorption equilibrium was established. In contrast, the photocatalytic reactions under UV–vis were very effective in comparison with the adsorption step. In the case of B/CNC/nanoSiO_2_ catalyst, the degradation percentage of MB reached a maximum of about 98% after 2 h of illumination. B/L-CNC/nanoSiO_2_ showed a very effective photocatalytic activity and reached about 93% of degradation percentage. However, C/CNC/nanoSiO_2_ and C/L-CNC/nanoSiO_2_ showed lower degradation percentages, 82 and 74%, respectively, compared to the two other catalysts. Nevertheless, they were considered important. The more excellent photocatalytic activity of B/CNC/nanoSiO_2_ and B/L-CNC/nanoSiO_2_ could be explained by their higher specific surface area compared to C/CNC/nanoSiO_2_ and C/L-CNC/nanoSiO_2_.

#### 3.3.5 Study of the Photocatalytic Degradation Kinetics

It is widely known that the photocatalytic degradation of organic molecule models follow first-order kinetics.^65^ Therefore, the kinetic models of MB degradation over different catalysts were studied under optimal conditions, applying the first-order kinetic model expressed using eq 4
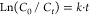
4where *C*_0_ and *C*_*t*_ represent the initial concentration of MB solution and solution concentration at time *t* (mg/L), respectively, *k* was the rate constant (min^–1^), and *t* was the reaction time (min).

Figure 9c represents the plot of Ln (*C*_0_/*C*_*t*_) versus UV–vis irradiation time (*t*) and which shows that the rates of reaction (*k*) were evaluated from the slope of the straight lines. The values of *k* were found to be 0.0184, 0.0135, 0.0112, and 0.0093 min^–1^ in the case of B/CNC/nanoSiO_2_, B/L-CNC/nanoSiO_2_, C/CNC/nanoSiO_2_, and C/L-CNC/nanoSiO_2_ catalysts, respectively. These first-order reaction rate constant values were higher than those reported in previous studies using other catalysts for degradation of MB.^61,66^ The experimental result showed that the coefficient correlations (*R*^2^) were higher than 0.98, proving that the photocatalytic degradation of MB was effectively well-described by a pseudo-first-order kinetic model.

#### 3.3.6 Photodegradation of the MB Mechanism

Photocatalytic degradation of the organic contaminants takes place when a semiconductor catalyst is irradiated with an UV-light source. Nanoscale semiconductor photocatalysts possess higher-surface area than their bulk counterparts and thus allows for greater photon absorption on the photocatalyst material surface. Furthermore, recombination of the electron–hole pair within the photocatalyst particle is drastically reduced as particle size decreases. When particle size of the photocatalyst decreases to the nanometer scale, the band gap energy greatly increases which in turn leads to higher redox potentials in the system. Hence, the nanoscale photocatalyst is expected to present higher photodegradation activity than that in its bulk.^67^

**Figure 10:**
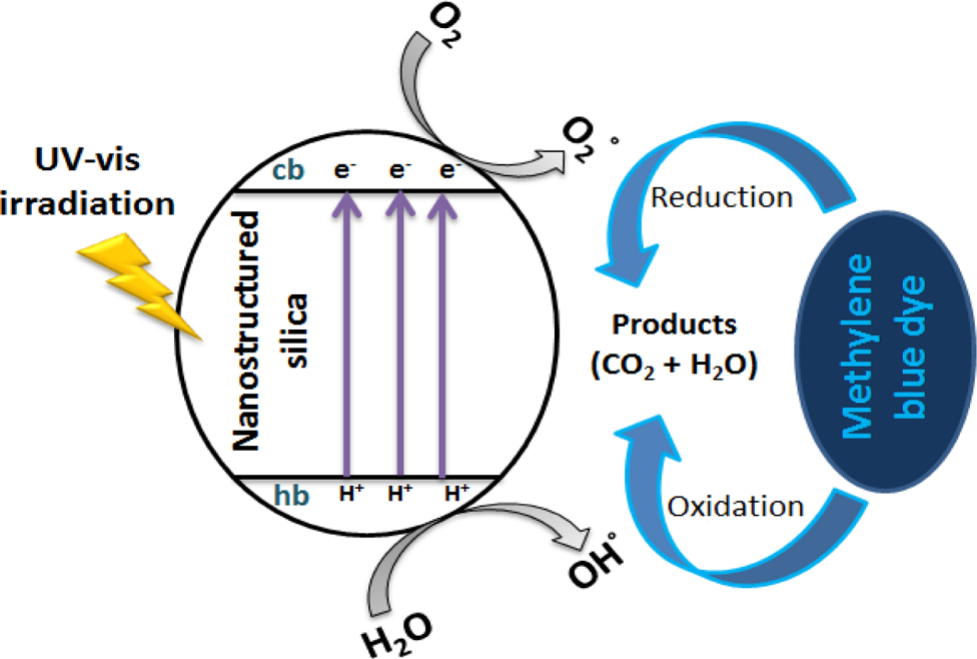
Photodegradation mechanism of MB by nanostructured silica.

In the present work and based on the obtained results and on previous studies,^68−70^ a probable photodegradation mechanism of MB by the synthesized nanostructured silica photocatalysts (nanoSiO_2_), which were acting as semiconductors, is illustrated in Figure 10.

It is well known that the charge transfer or photo-generated electron migration (*e*^–^) occurs from the valence band (VB) to conduction band (CB), overcoming the band gap of the excited photocatalyst upon UV–vis irradiation (*h*ν). When the nanostructured hollow silica (nanoSiO_2_) nanoparticles are suspended in solution, they absorb energy in the range of their band gaps, UV or near-UV photons, an electron of the VB is transferred to the CB, forming a positive hole in the VB.
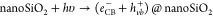
5

In general, three possible reaction pathways have been proposed for the photochemical reaction

(1) dye molecule is directly oxidized by a positive hole forming a cation radical, which reacts rapidly with oxygen
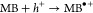
6
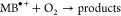
7

(2) Water trapped the  onto the nanoSiO_2_ surface to generate protons (H^+^) and hydroxyl radicals (OH^•^), where OH^•^ species acts as an efficient oxidizing agent to decompose MB molecules to smaller species, such as CO_2_ and H_2_O.
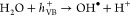
8
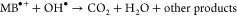
9

(3) The free electrons of the CB  would produce free anionic superoxide radicals of oxygen (O_2_^–•^) through the reaction with dissolved oxygen (O_2_).
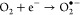
10

The superoxide anion radical (O_2_^–•^) may be further reduced to hydrogen peroxide (H_2_O_2_), according to the following equations
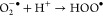
11
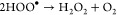
12
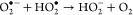
13
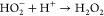
14

The created H_2_O_2_ can be dissociated into 2^•^OH radicals, leading to the enhanced MB molecule degradation rate.
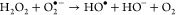
15
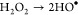
16
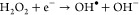
17

At pH 10, increasing hydroxyl group concentration may improve the photocatalytic degradation efficiency, where OH^–^ groups can be reacted with h^+^ to produce numerous ^•^OH and H^+^ ions.

For the proposed mechanism mentioned above, the presence of H_2_O_2_ during the photocatalytic degradation of the MB dye was confirmed. For this purpose, KMnO_4_ was used as the titrant. The dark purple color of permanganate solution diminished when it was added to the reaction medium; this is consistent with the production of H_2_O_2_ during the photocatalytic decomposition of dye. The permanganate ion, acting as an oxidizing agent, leads to the oxidation of H_2_O_2_ that is oxidized to oxygen gas. The permanganate ion, in turn, is reduced from the +7 oxidation state in  (purple) to the +2 oxidation state in Mn^2+^ (no color).
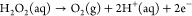
18
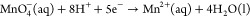
19

## 4 Conclusions

Lignin-containing and lignin-free cellulose II nanocrystals (L-CNCs and CNCs) were successfully extracted by chemo-mechanical treatments from abundant renewable hardwood and softwood sawdusts (Cedar and Beech). The findings of our study have confirmed that the properties of the nanocellulose were highly dependent on the nature of the raw materials under the same experimental conditions. L-CNCs and CNCs extracted from purified Beech sawdust exhibit average lengths of about 150 nm and diameters less than 9 nm. However, in the case of Cedar source, the obtained L-CNCs and CNCs presented average lengths around 90–100 nm and diameters around 10 nm. With such interesting characteristics and high crystallinity index, both L-CNCs and CNCs have been applied as organic templates for the synthesis of nanostructured silica. With comprehensive analysis by SEM, TEM, size distribution, XRD, FTIR, TGA/DTA, and nitrogen sorption analysis, the prepared nanostructured silica materials were found to be quite uniform and coined the shape of L-CNCs and CNCs with high specific surface areas, which reached around 950–1100 m^2^/g for B/L-CNC/nanoSiO_2_ and B/CNC/nanoSiO_2_. Correspondingly, the surface areas were less, around 350–400 m^2^/g for C/L-CNC/nanoSiO_2_ and C/CNC/nanoSiO_2_, respectively. Thereby, the application of these nanocrystals was beneficial to controlling the spatial arrangement of the porous nanostructured silica materials. Thus, they are found to be promising materials as catalysts for photocatalysis degradation of MB dye because of their large specific surface areas. Significant degradation rates of MB dye were determined to reach higher than 90% for B/CNC/nanoSiO_2_ and B/L-CNC/nanoSiO_2_ and around 70–80% for C/CNC/nanoSiO_2_ and C/L-CNC/nanoSiO_2_, respectively. Both lignin-containing and lignin-free CNCs extracted from softwood and hardwood sawmill wastes have proven to be promising biotemplates for preparing nanostructured porous materials used as alternative catalysts to degrade the organic cationic dye.

## Supporting Information Available

Experimental details for the preparation and the characterization methods of Kraft pulps, bleached fibers, L-CNCs, CNCs, and nanostructured hollow silica; morphological and structural analyses of raw sawdusts, cooked, and bleached pulps by SEM, FTIR, and XRD; morphological analysis of CNCs and L-CNCs; plots of particle size distribution of CNCs and L-CNCs; and plots of EDS, particle size distribution, and BET analysis of nanostructured hollow silica (PDF)
