# Supplementary material for: Lignocellulosic Nanocrystals from Sawmill Waste as Biotemplates for Free-Surfactant Synthesis of Photocatalytically Active Porous Silica
Source: ACS Appl Mater Interfaces. 2022 Apr 20;14(17):19547–60. doi: 10.1021/acsami.2c02550 (PMC9073848; doi:10.1021/acsami.2c02550)
Supplement: Supplementary file 1 — am2c02550_si_001.pdf [file am2c02550_si_001.pdf]

Supporting Information to ACS applied materials & interfaces

Lignocellulosic Nanocrystals from Sawmill Waste as Bio-Templates for Free-Surfactant Synthesis of Photocatalytically Active Porous Silica

Maryam El Hajam[1,2,3,*], Noureddine Idrissi Kandri[2], Abdelaziz Zerouale[1], Xiaoju Wang[3,4], Jan Gustafsson[3], Luyao Wang[3], Ermei Mäkilä[5], Leena Hupa[6] and Chunlin Xu[3]

*Corresponding authors: maryam.elhajam@usmba.ac.ma (M. El Hajam)

1. Processes, Materials and Environment Laboratory (PMEL), Faculty of Sciences and Techniques, Sidi Mohammed Ben Abdellah University, Road Imouzzer, BP 2202, Fez, Morocco

2. Signals, Systems and Components Laboratory (SSCL), Faculty of Sciences and Techniques, Sidi Mohammed Ben Abdellah University, Road Imouzzer, BP 2202, Fez, Morocco

3. Laboratory of Natural Materials Technology, Åbo Akademi University, Henrikinkatu 2, Turku FI-20500, Finland

4. Pharmaceutical Sciences Laboratory, Faculty of Science and Engineering, Åbo Akademi University, Tykistökatu 6A, Turku FI-20520, Finland

5. Laboratory of Industrial Physics, Department of Physics and Astronomy, University of Turku, Turku FI-20520, Finland

6. Laboratory of Molecular Science and Technology, Åbo Akademi University, Henrikinkatu 2, Turku FI-20500, Finland

I. Materials and methods

I.1. Delignification using alkaline pulping

The pre-prepared sawdust fibres from softwood and hardwood were first subjected to an alkaline pulping treatment (cooking) in order to remove hemicellulose, lignin, and other impurities by saponification and cleavage of lignin−carbohydrate linkages. This delignification stage was accomplished using an alkaline leaching of NaOH/Na2S. The mixture was transferred into a 10-liter electrically heated and thermostatically controlled rotary digester with a heating jacket and a special agitation system (cooking reactor) containing 500 g of each sawdust separately. The amount of cooking liquor was calculated and 25% alkali charge was used for the pulping processes based on the oven dry weight of raw material. The percentage of Na_2S used was 0.1%. The considered factors were: cooking time of 45 min, temperature 165 ◦C, and liquid/solid ratio of 10. After the alkali treatment process, the insoluble residue (black-brown fibers) were filtered and then washed several times using distilled water in a hydra-pulper to eliminate completely the black liquor from pulp and the moisture content was measured. The obtained cooked pulp (Kraft pulp) was freeze-dried for 73 h prior any further analysis.

I.2. Bleaching process

Although, the most of the lignin and hemicellulose was removed during the alkaline pretreatment step, the remaining part of non-cellulosic compounds was removed using the bleaching method. To do this, a part of each dried cooked pulp was separately subjected to a bleaching process by adding equal parts of buffer solution (27 g NaOH and 75 ml glacial acetic acid, diluted to 1 L of distilled water) and aqueous chlorite (1.7 wt % in water). The mixture was being boiled at 80 °C using a silicon oil bath for 2 h under mechanical stirring (fiber to liquor ratio of 1:20). After this treatment, the bleached fibres were allowed to cool down, and were subsequently filtered and washed thoroughly using excess distilled water until the pH of the filtrate was neutral. The bleached fibers were pure white in color. Finally, the obtained cellulose was freeze-dried for 72 h and stored in polyethylene bags until its use.

I.3. Lignocellulose and cellulose nanocrystals isolation

The cooked and bleached pulps were subjected to a mechanical and acid hydrolysis process to obtain colloidal suspensions of lignin-containing cellulose nanocrystals (L-CNCs) and lignin free

cellulose nanocrystals (CNCs) respectively. These processes were performed to each Kraft and bleached pulps extracted from Cedar and Beech sawdusts for the first time.

10 g of each dried samples were first milled with a blender and then treated with preheated H_2SO_4 (64 wt %) with fiber to solution ratio of 1:20 (g/ml) at 45 °C under strong and constant stirring for 40 min. At the end of the reaction time, the hydrolysis was quenched immediately by adding 10-fold cold distillated water, the resulting mixture was then cooled down to the room temperature. The obtained white cloudy suspension was washed with Milli-Q water by successive centrifugations (Sorvall RT7 Plus) at 3800 rpm and 10°C for 20 min for each step until supernatant reached a pH value near to 3. Afterwards, the precipitate of the L-CNCs and CNCs suspensions were re-suspended in purified water and dialyzed for a week against purified water at room temperature in a dialysis membrane (with a width of 10 mm) to guarantee elimination of non-reactive sulfate groups, salts, soluble sugars and different compounds that could be formed during the hydrolysis. Subsequently, the suspension was dispersed and homogenized using high-pressure nano-homogenizer (ATS AH-100D, China) by one pass at a pressure of 600 bar and two passes of 1000 bar to obtain homogeneous suspension and remove the agglomerates by breaking down the unfractionated cellulose into small nano-metric particles in order to form homogeneously dispersed suspension. The resulting suspensions were stored in a refrigerator at 4 °C for further analyses or utilization. The homogenized suspension of L-CNCs and CNCs underwent freeze-drying to perform additional characterizations. The denotations of obtained L-CNCs and CNCs samples based on different cellulose sources are: C/L-CNC, C/CNC, B/L-CNC and B/CNC.

I.4. Physicochemical characterization

a. Scanning Electron Microscopy-Energy Dispersive Spectroscopy (SEM-EDS)

The surface morphology of raw, cooked and bleached fibres obtained at the different steps of the chemical processes as well as the elemental composition of nanostructured porous silica were evaluated by scanning electron microscope-energy dispersive spectroscopy (SEM-EDS), using a LEO Gemini 1530 SEM (Germany). Each sample was dried and placed in metallic holder, and coated by a thin layer of carbon to promote the conductivity and analyzed using an accelerating voltage of 5 kV.

b. Transmission Electronic Microscopy (TEM)

The morphological properties (structure and size distribution) of lignocellulose and cellulose nanocrystals isolated from each cooked and bleached pulp-fiber successively as well as the nanostructured silica were examined by transmission electronic microscopy (TEM), using a JEM-1400 Plus TEM, JEOL Ltd., (Japan). The homogenized L-CNCs, CNCs and nanostructured porous silica suspensions were diluted to 0.01 % by mixing with Milli-Q water under ultrasonication for 10 min. About 20 µL of each diluted suspension were dropped and deposited separately onto a carbon coated copper grid using a pipette, the grid was air dried at room temperature for 3 min and the excessive water was drained with a filter paper. Then the grid was negatively stained with a 2 % (w/w) solution of uranyl acetate and dried at room temperature for 1 min in the case of L-CNCs and CNCs samples, the redundant liquid was drawn away using a filter paper also before the TEM analysis with an accelerating voltage of 80 kV.

The dimensions of whiskers (lengths and width) were determined using digital image analyses (ImageJ). A hundred nanorods were randomly selected and a minimum of 100 measurements were used to determine the average length and the diameter, respectively.

c. Particle size distribution and zeta potential measurement

The particle size distribution and the surface charge of the particles (zeta potential) of the L-CNCs and CNCs and nanostructured silica suspensions were measured using a Malvern Zetasizer 3000 (Malvern, United Kingdom). For this measurement, a capillary cell was used and the L-CNCs, CNCs and nanostructured porous silica suspensions were diluted to a concentration of 0.01 % (w/v) with Milli-Q water and sonicated in an ultrasonic bath for 30 min before being analyzed. Each sample was tested in triplicate, while 15 runs were performed for every test, and the average values were reported.

d. Fourier Transform Infrared (FTIR) Spectroscopy analysis

Untreated, alkali-treated, bleached, and acid-hydrolyzed fibres samples as well as silica-coated L-CNCs, CNCs and nanostructured silica were analysed by FTIR using a Nicolet iS50 FTIR spectrometer (USA) equipped with a Specac Golden Gate single-reflection ATR (attenuated total reflection) accessory, in order to examine any changes in functional groups during each treatment in the samples. The chemical groups for each freeze-dried and finely grounded sample were analyzed at 2 cm^{-1} resolution within the scanning range of wavenumber 400–4000 cm^{-1} and 32

accumulated scans were acquired and co-added in order to achieve an acceptable signal-to-noise ratio. OMNIC 4.0 software was applied to track the significant peaks transmittance positions at a particular wavenumber, the spectra were recorded in transmittance band mode.

e. X-ray diffraction (XRD) analysis

The raw, cooked, bleached and hydrolyzed samples as well as silica-coated L-CNCs, CNCs and nanostructured silica were subjected to a XRD analysis using an X-ray diffractometer (Bruker Discover D8, Germany) (wavelength of 1.5406 A). Each dried material in the form of milled powder was pressed into flattened sheets on a sample holder to obtain total and uniform X-ray exposure. Samples were scanned at room temperature with monochromatic Cu Kα radiations source (λ=0.15406 nm) operated at voltage of 40 kV and current of 30 mA. XRD data were collected within a diffraction angle (2θ) range of 10°–40° at scanning speed of 2 °C/min.

f. Differential thermal analysis and thermogravimetric analysis (DTA/TGA)

A TA Instruments SDT Q600 Simultaneous Thermogravimetric Analyzer was used to characterize the thermal stability of the L-CNCs, CNCs, silica-coated L-CNCs or CNCs and nanostructured silica samples (measuring the mass transformation as a function of temperature). Approximately 5-10 mg of each sample were heated from room temperature (=23 ◦C) to 700 °C at a heating rate of 10 °C/min using alumina crucibles without lid. All of the measurements were performed under air atmosphere.

II. Results and discussion

II.1. Preparation of cellulosic fibres

- ***Morphological analyses of raw, cooked and bleached pulps***

Figure S1 illustrates the visual macroscopic changes of the Cedar and Beech sawdusts after each step of the treatment. The color of raw Cedar and Beech fibers is more or less brown and changes to dark beige after the alkaline modification. Contrary to the cooked sawdusts, the bleached pulps are obviously different and look perfectly white. These changes in color are owing to the removal of non-cellulosic compounds and other impurities such as lignin, hemicelluloses, pectin, and wax during the chemical treatments of the wood sawdusts. The white colour of the end product is a characteristic of the pure cellulosic fibers.

These results confirmed the obtained yields of pulps after alkali and bleaching stages of both Cedar and Beech sawdusts in regard to the initial weight of raw and cooked fibers successively. These yields reached 50 % and 95 % for cooked and bleached pulps extracted from Beech sawdust respectively and 38 % and 89 % for those extracted from Cedar sawdust respectively. The low yields of cooked pulps confirm the dissolution of the majority of lignin and hemicellulose during the alkali treatment.

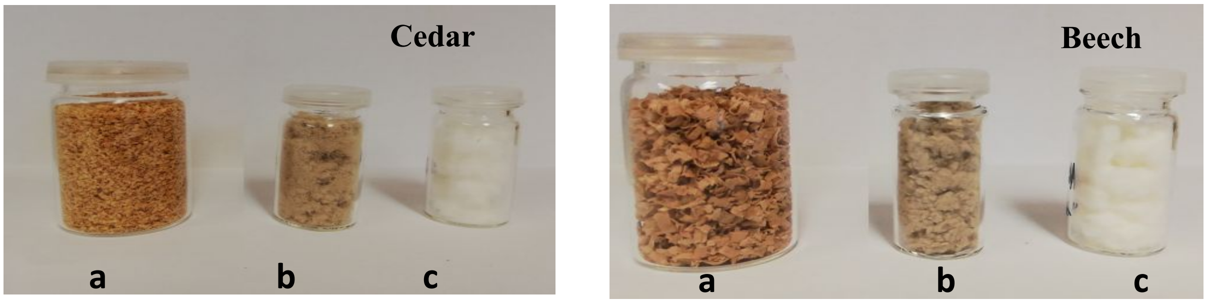

Figure S1: Photographs of Cedar and Beech sawdusts (a) untreated, (b) alkali-treated and (c) bleached fibers

- ***Scanning Electron Microscopy (SEM)***

The morphological surface of raw (A), cooked (B) and bleached (C) sawdust fibers was elucidated using scanning electron microscopy (SEM), in order to evaluate the influence of each treatment step over the lignocellulosic structure of softwood and hardwood sawdusts and to assess the apparent shape of crude sawdusts and the changes occurred after each treatment.

SEM micrographs of untreated sawdusts (Figure S2- A) display lot of non-fibrous components (waxes and pectins) covering the surface of the fibers. These fibers are oriented and bonded by hemicelluloses, lignin and other water-soluble components playing the role of binders. The overall surface of the raw fibers is found to be somewhat smooth due to the presence of waxes and oil. The micrograph of Cedar sawdust (softwood) (Figure S2- A1) exhibits multicellular and porous morphology and shows horizontal tubes known as tracheids; the thickened walls of the tracheids are marked by lines of minor and major holes, known as punctuations [1]. The morphological examination of Beech sawdust (hardwood) (Figure S2- A2) shows the presence of elongated tight fibers which play only the supporting role and the vessels that provide the conduction [1].

After alkali treatment step, the SEM micrographs (Figure S2-B) show roughness in the surface of the cooked fibers. The wood sawdusts were defibrillated into small bundles of fibers and individual fibers start to separate indicating the beginning of the appearance of the bundle cellulosic fibers due to partial removal of the outer non-cellulosic compounds contained in the wood sawdusts (cementing materials) [2,3]. The alkali treatment may causes the degradation of some alkali-labile linkages (ester and ether linkages) and glycosidic side chain between lignin monomers or between lignin and polysaccharides, resulting mainly in structural alteration of lignin, cellulose swelling, and solvation of hemicellulose, which helps in the partial defibrillation of fibers and subsequently improves the penetration of the bleaching solution during bleaching treatment [4]. Images (C1 and C2) present the micrographs of bleached Cedar and Beech sawdust succivelly. The presence of chlorine during the bleaching step allows the oxidation of the remaining lignin in the cooked pulps and generates hydroxyl, carbonyl and carboxylic groups which permit the solubilization of residual lignin [5–7].

The size of fibers varies over a broad range, which may be attributed to the uneven distribution and density of the adsorbed hemicellulose chains. The diameter of the woody material decreased, indicating that under the strong chemical treatment conditions all the components that bind the fibril structure of wood sawdusts were removed, hence allowing the fibres to separate into an individual form. This fact confirms the removal of an important part of the amorphous cementing components between the fibers during the treatment stages.

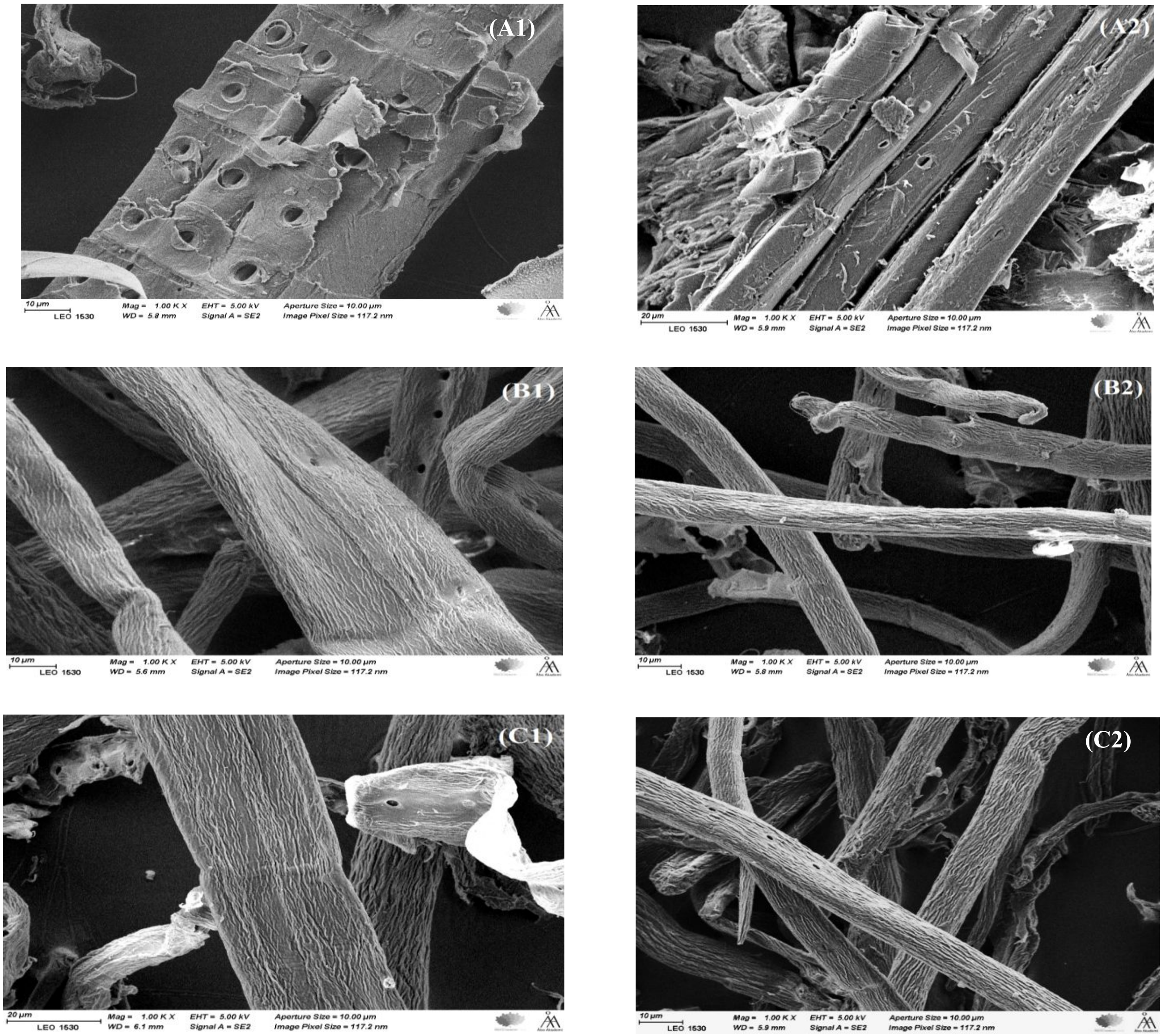

Figure S2: SEM images of untreated Cedar (A1) and Beech (A2), alkali-treated Cedar (B1) and Beech (B2) fibres, and bleached Cedar (C1) and Beech (C2) fibres

- ***Fourier Transform Infrared (FTIR) Spectroscopy analysis***

FTIR spectroscopy is an efficient and relatively easy technique extensively used in cellulose research; it aims to monitor the chemical structural changes that occurred during the entire isolation process and to evaluate the structure of each obtained material by identifying the functional groups present in their surfaces [8]. The recorded spectra of raw, cooked and bleached pulps extracted from Cedar and Beech sawdusts are shown in Figure S3.

It is clearly observed that the spectra of both treated pulps (cooked and bleached) present differences compared to those of the raw samples in both cases (Cedar and Beech sawdusts). These differences manifested in the disappearance of some characteristic bands assigned to non-cellulosic compounds such as hemicelluloses and lignin [9]. As the main constituents in wood sawdust are cellulose, hemicellulose and lignin, the spectra of both raw sawdusts (Figure S3- a) showed markedly the typical band patterns of these lignocellulosic compounds.

As can be seen, FTIR spectra of all untreated and treated samples from Cedar and Beech sawdusts have exhibited broad and dominant peaks between 3330-3440 cm^{-1} which are attributed to the free O-H stretching vibration of the OH groups in cellulose molecules, while the relative weak bands around 2890 cm^{-1} correspond to saturated C-H stretching vibrations from methyl (CH) and methylene (CH_2) groups (in cellulose, hemicellulose and lignin) respectively. The intensity of theses peaks increases after cooking and bleaching processes for both Cedar and Beech sources confirming the removal of non-cellulosic materials [10–12]. The small band centered at 1737 cm^{-1} in the spectrum of raw Cedar and the prominent band around 1743 cm^{-1} in that of raw Beech samples are predominantly attributed to C=O stretching vibration of the acetyl and uronic ester groups, from pectin, hemicellulose or the ester linkage of carboxylic group of ferulic and p-coumaric acids of lignin and/or hemicellulose. These peaks disappears completely upon chemical treatment of both sawdusts (Cedar and Beech) confirming the removal of the most hemicellulose and lignin after cooking and bleaching treatments [13–16]. The peaks at 1640 cm^{-1} present in the all cooked and bleached samples were ascribed to O-H bending vibration of the adsorbed water, indicating the strong interaction between cellulose and water [17–20]. The absorption peaks observed at 1602 cm^{-1}, 1449 cm^{-1} in both spectra of raw Cedar and Beech and those appeared at 1508 cm^{-1} in the spectrum of raw Cedar and at 1515 cm^{-1} in that of raw Beech are representative of the C=C bond elongation of the lignin aromatic ring, and symmetric CH_2 bending vibration, the intensity of all these peaks decreased after the alkali treatment, whereas they have completely disappeared after the bleaching stage, and further indicating the partial and the totally removal of lignin during the cooking and bleaching treatment successively [13,16].

Likewise, the three common absorption bands observed at 1421, 1364, 1314 cm^{-1} were mainly assigned to CH_2 symmetric bending, C–O symmetric stretching and C-H bending of cellulose respectively, the intensity of these bands increase after chemical treatment confirming the extraction of typical cellulose [21]. While the peak around 1264 cm^{-1} present only in the spectra

of raw sawdusts corresponds to guaiacyl ring breathing, C–O out of plane stretching vibration of the aryl group in lignin C-O linkage in guaiacyl aromatic methoxy groups [16,22,23]. This band significantly decreased after chemical treatments, confirming also the removal of most lignin during the chemical treatments.

Furthermore, the obtained spectra showed a slight increase in the bands intensities observed between 1157 and 500 cm^{-1} after chemical treatments, proving the removal of hemicellulose and lignin species [24]. The prominent absorption bands found at 1157, 1022 are attributed to C–O–C asymmetric stretching and C−O−C pyranose ring skeletal vibration of the cellulose molecule successively, these peaks became sharper and narrower and their intensities were gradually increased from untreated sawdusts to cellulose fibers as the cellulose content of the treated fiber getting increased during the chemical treatments [16,24,25]. Typically, the structure of cellulose II was confirmed by the enhancing of the intensity of the peak observed at 1022 cm^{-1} [20]. Additionally, the band at 894 cm^{-1} was assigned to the glycosidic C-H deformation with ring vibration contribution and OH bending, which is characteristic of glycosidic linkages between glucoses in cellulose [26,27], the growth of this peak showed the increase in the percentage of cellulosic components. The band observed at 664 cm^{-1} is attributed to the out of plane deformation of C−H functional group, and the peak at 611 cm^{-1} indicates C-OH out of plane bending vibration [28].

These results indicate that the cellulose component was not removed throughout the chemical treatments preformed on Cedar and Beech sawdusts, it is the common product found in all samples. However, FTIR spectra agree with the effective removal of hemicellulose and lignin during the purification process.

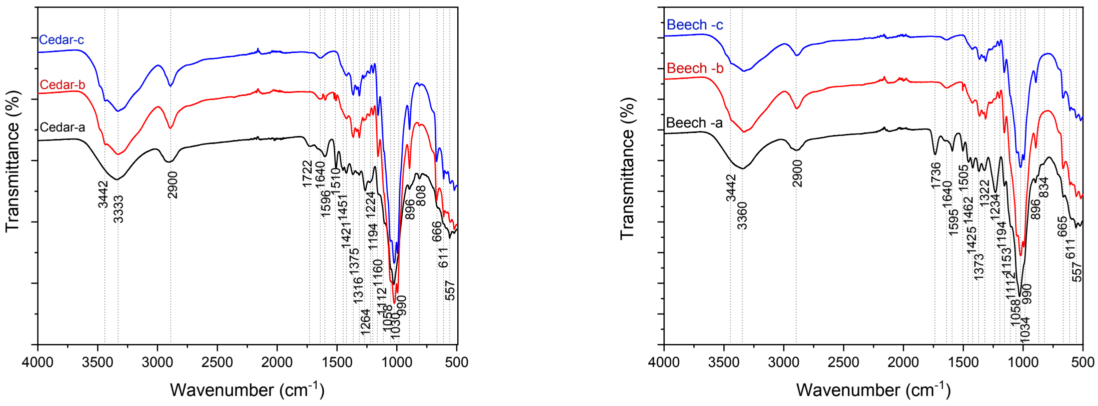

Figure S3: FTIR spectra of Cedar and Beech fibers successively: a) raw, b) cooked and c) bleached

- ***X-ray diffraction (XRD) analysis***

Contrary to hemicelluloses and lignin which are totally amorphous polymers, cellulose has both amorphous and crystalline states in nature; according to Zhang and Lynd [29], the crystalline fraction of cellulose is formed due to the hydrogen bonding interactions and Van der Waals forces between adjacent molecules. X-ray diffraction (XRD) analysis was carried out to evaluate and follow the evolution of crystallinity of the Cedar and Beech sawdusts before and after different chemical treatment stages.

Figure S4 shows the diffraction patterns of raw, alkali treated and bleached Cedar and Beech sawdusts successively. The crystallinity index and crystallites diameters values were presented in table S1.

The diffractograms of both raw Cedar and Beech materials display three dominant peaks (hump) at 2θ values corresponding to 16°, 22° and 34°, that are characteristic of native crystalline structure e.g. cellulose I of natural fibers [30–34]. These peaks are attributed to the diffraction plans (10-1) of amorphous cellulose and (002) and (040) of the crystalline form respectively.

After alkali treatment, clear significant changes were observed in the peak positions of both cooked Cedar and Beech pulps. These observations indicate that a polymorph conversion of cellulose I to cellulose II has occurred throughout the cooking stage under the applied conditions [35,36]. As it is seen from the diffractograms of cooked pulps from both Cedar and Beech sawdusts, three well-defined and higher intensity peaks appeared and dominated at 2θ around 12°, 20° and 22°, had Miller indices of (101), (10-1) and (002) successively, indicating the crystallographic form of cellulose II as shown in literature [37,38]. In the case of cooked pulp originated from Beech sawdust, we noticed the appearance of two other weak peaks at 2θ=15.1° and 16.5°, which correspond to the (101), (10-1) assigned to cellulose I, which is still remain after the alkali treatment, these later were resulted by the breakup of peak at 2θ= 16.21° characteristic of cellulose I in raw Beech. However, when the source material was Cedar sawdust, the cooked pulp displays only the characteristic profile of cellulose II after alkali treatment. All these peaks appeared after the alkali treatment in both Cedar and Beech cases remain present upon bleaching step and become more defined as expected, because of the progressive removal of all non-cellulosic compounds after the bleaching treatment [30,34]. This finding is further confirmed by measuring the crystallinity index (CrI) of all studied samples.

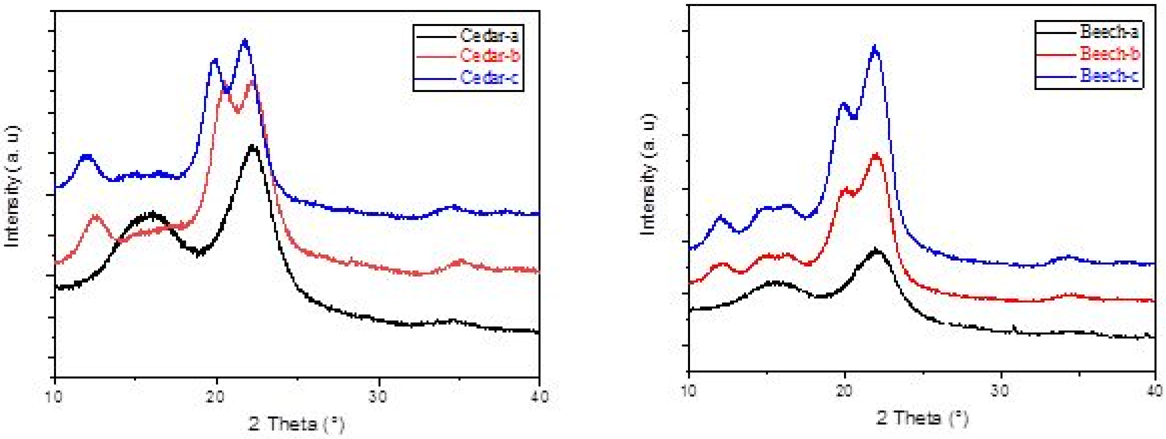

Figure S4: XRD spectra of Cedar and Beech fibers successively: a) raw, b) cooked and c) bleached

The crystallinity results (table S1) showed that both raw Cedar and Beech presented the lower crystallinities, which are about 41.24 and 40.83 % successively when compared to their cooked or bleached forms. A progressive and continuous increase has been noticed in cristanility index (CrI) and crystallite diameters (D) upon the successive chemical treatments, from the alkaline to the bleached pulps for both Cedar and Beech sawdust, with lower values for Cedar (softwood). The crystallinity of bleached Beech is higher than that of bleached Cedar due to the dense structure, lower lignin and hemicellulose content of cellulose hardwood fibers [39].

It is assumed that the extracted bleached pulps from Cedar and Beech are composed of pure cellulose and its CrI were measured at 74 % and 76 % successively, which are higher than that measured for the raw samples, indicating that non-cellulosic compounds were totally removed after the alkali and bleaching treatments [9]. Indeed, the applied alkali treatment was effective to partially remove such non-cellulosic compounds, because the measured CrI of the alkali treated was found to be lower than that of bleached pulps and higher than that of raw sawdusts. Similar results were also reported by R. Moriana et al. [40] who observed an increase in crystallinity index after extraction of cellulose from different forests residues [41]. This increase could be attributed to the removal of amorphous lignin and hemicelluloses as well as the rearrangement of cellulose chains [42,43].

Table S1: Crystallinity index and crystallites sizes of Cedar and Beech before and after the different treatments

Samples	Cedar			Beech		
	Raw	Cooked	Bleached	Raw	Cooked	Bleached
**CrI (%)**	41.24	71.49	73.99	40.83	71.37	76.14
**D (nm)**	3.09	3.76	3.95	2.94	4.19	5.40

II.2. Isolation of lignocellulose and cellulose nanocrystals

- **Morphological analyses of lignocellulose and cellulose nanocrystals**

The nanocrystal suspensions (L-CNCs and CNCs) extracted from both cooked and bleached pulps of Cedar (softwood) and Beech (hardwood) sawdusts remained stable and did not reveal any phase separation or precipitation, showing that the obtained nanocrystals are perfectly dispersed and their size features are within the colloidal dimension range. The dark color of the L-CNCs suspensions could be certainly assigned to the presence of residual lignin (Figure S5).

As it is known, the extraction of nanocrystals using sulfuric acid hydrolysis was expected to induce grafting of negatively charged sulfate groups on the nanocrystal surface, resulting in the stabilization of the colloidal suspensions through repulsive interactions and making them well dispersed. After acid hydrolysis, the size of the fibres would be clearly reduced from the micron to the nanometer scale.

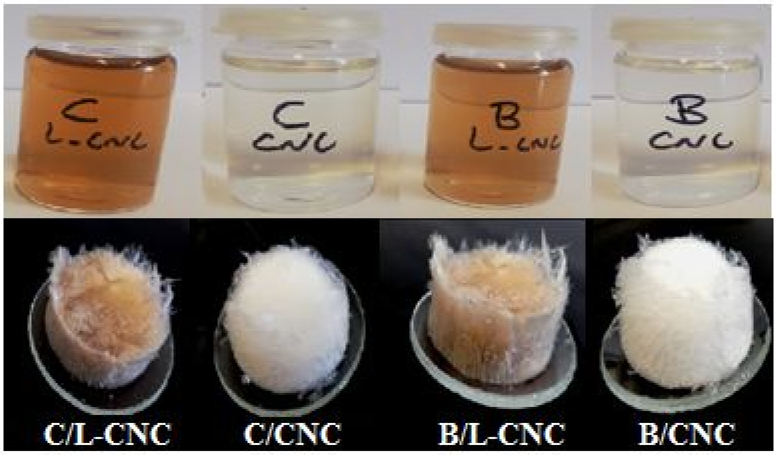

Figure S5: Photographs of liquid suspensions and freeze dried L-CNCs and CNCs extracted from Cedar and Beech sawdusts

III.2. Particle size distribution

Figure S6 presents the length and width distribution of L-CNCs and CNCs based on TEM and Imagej software analysis.

Figure S7 presents the particles size distribution of L-CNCs and CNCs from Cedar and Beech sawdusts obtained by DLS analysis.

III.3. Synthesis of nanostructured hollow silica

- **Energy-Dispersive Spectroscopy**

Energy dispersive spectroscopy analysis (EDS) proved that the coating consisted of Si and O (Figure S8).

- **Particles size distribution**

Figure S9 presents the particles size distribution of the different nanostructured hollow silica prepared by the intermediate of L-CNCs and CNCs cores from Cedar and Beech sawdusts obtained by DLS analysis.

Figure S10 presents the inner diameter distribution of different hollow nano-silica based on TEM analysis and Imagej software analysis.

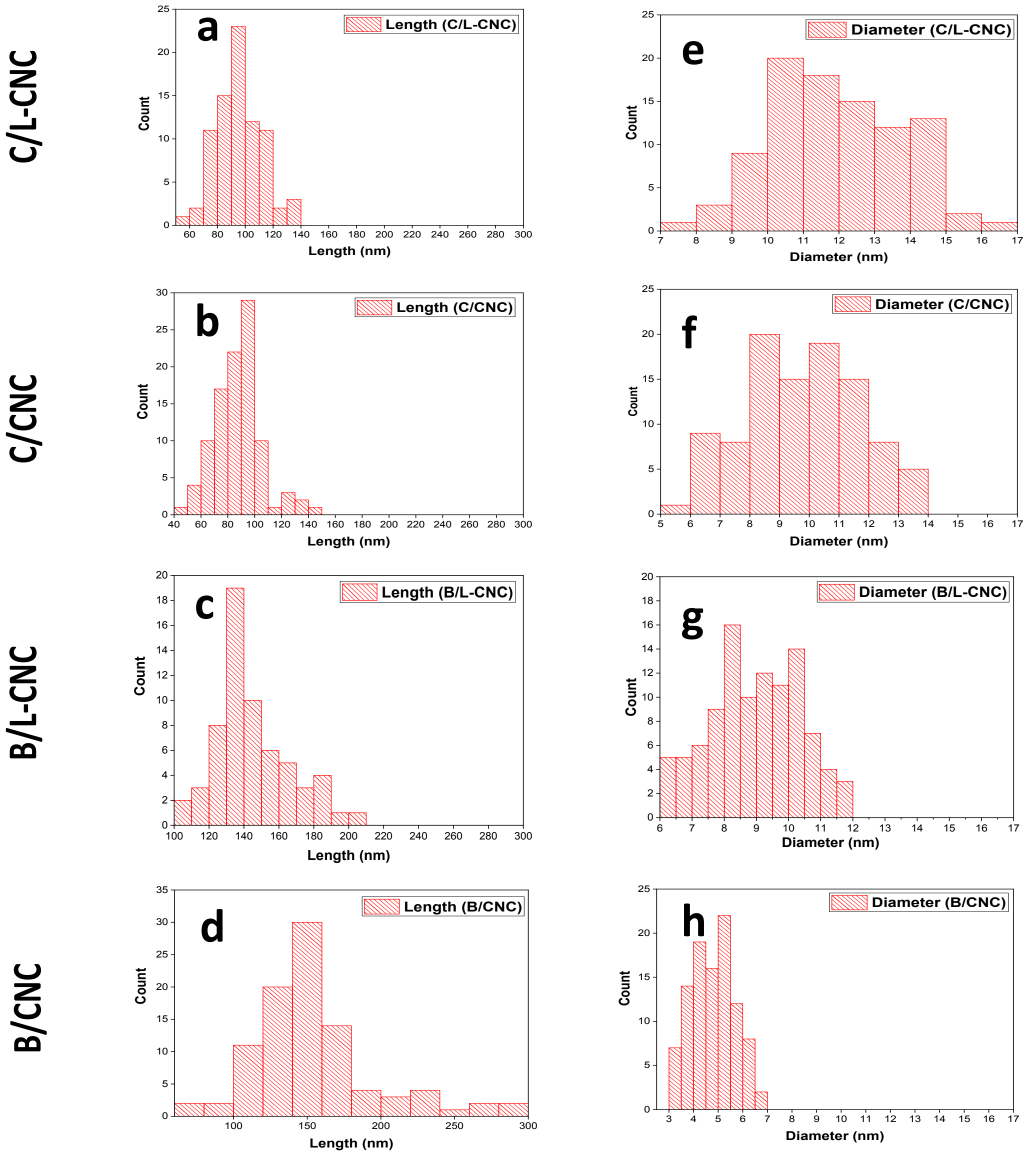

Figure S6: length (a-d) and width (e-h) distribution of L-CNCs and CNCs

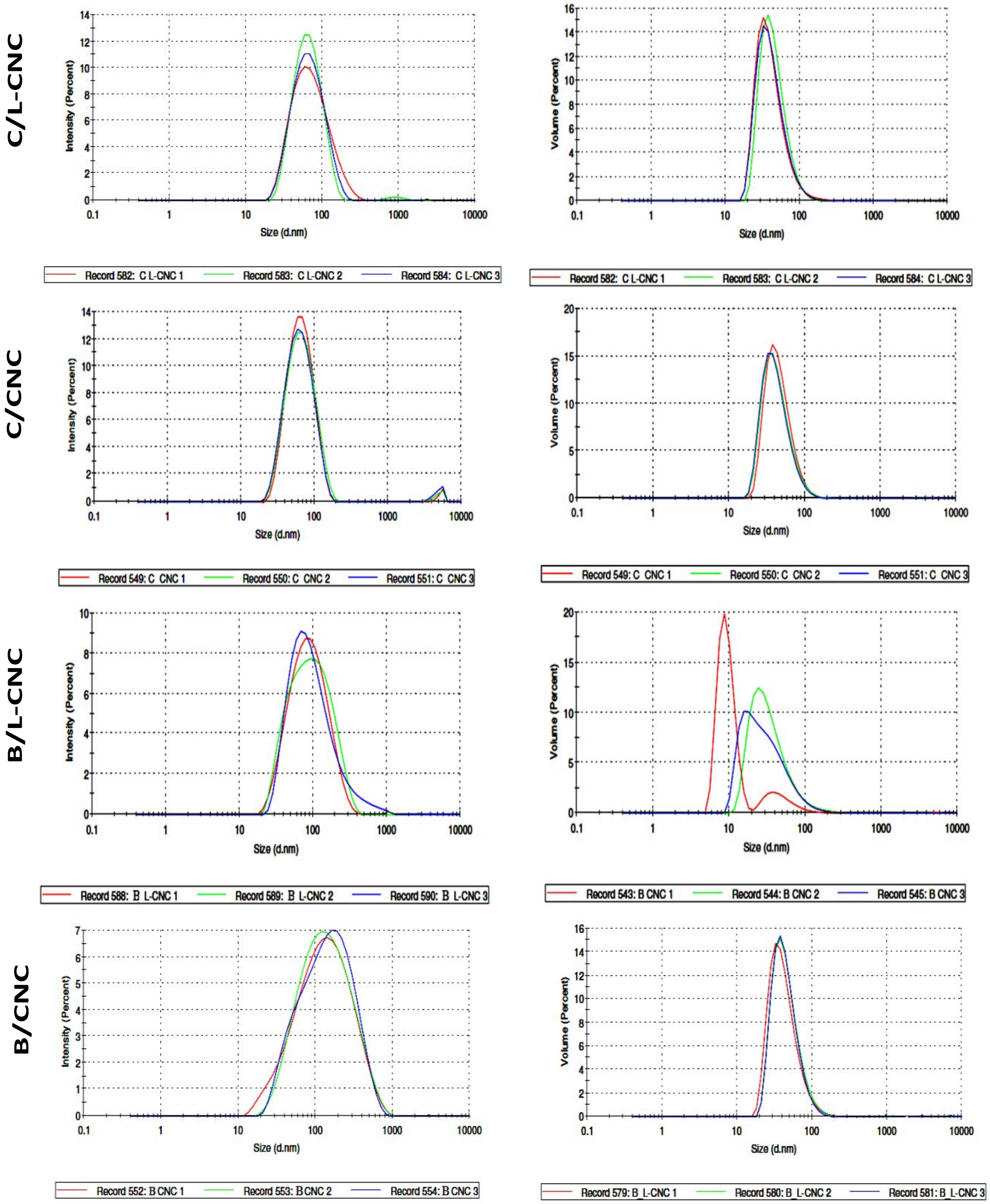

Figure S7: Particle size distribution of L-CNCs and CNCs extracted from Cedar and Beech sawdusts

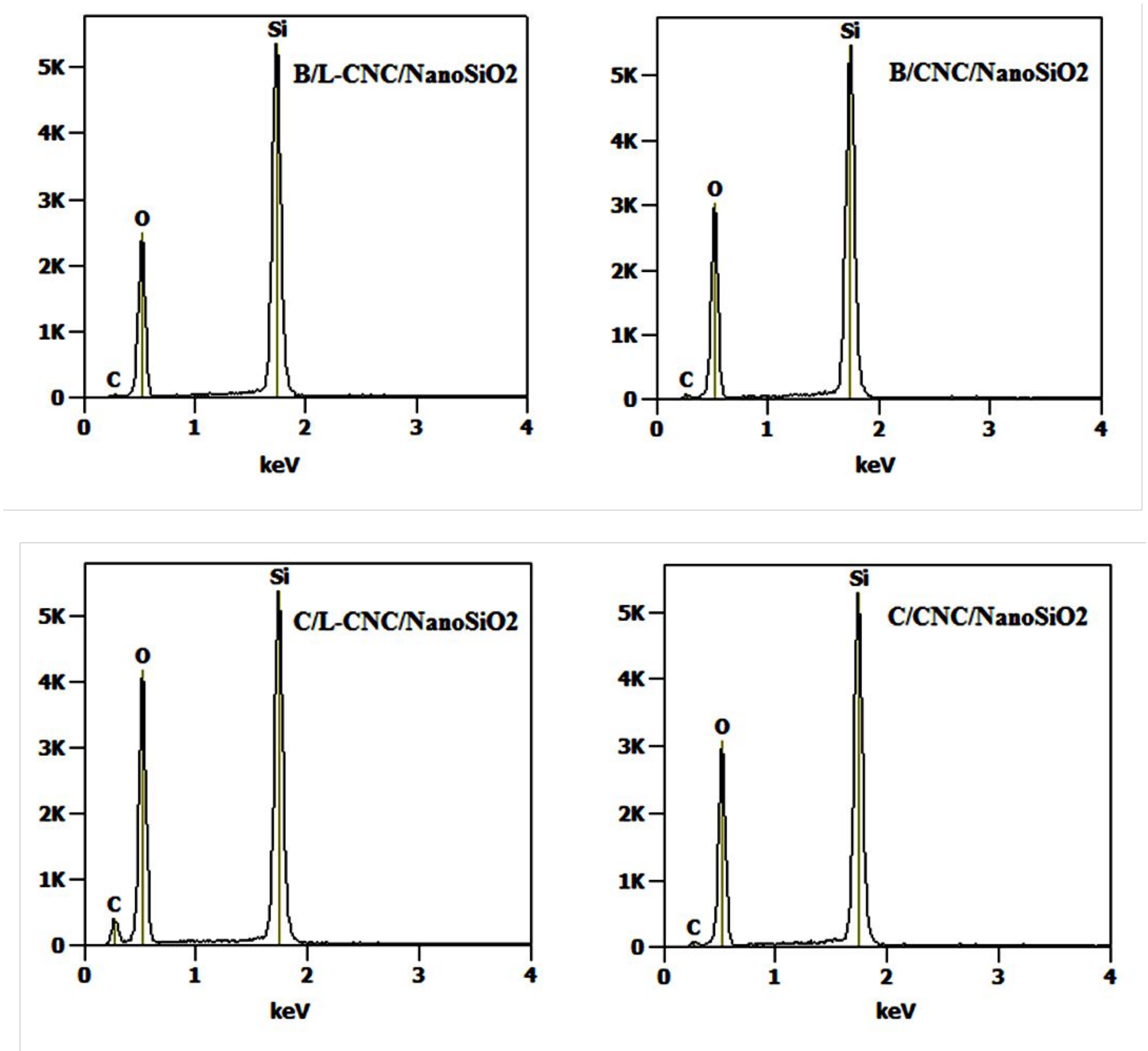

Figure S8: EDS analysis of different nanostructured porous silica materials

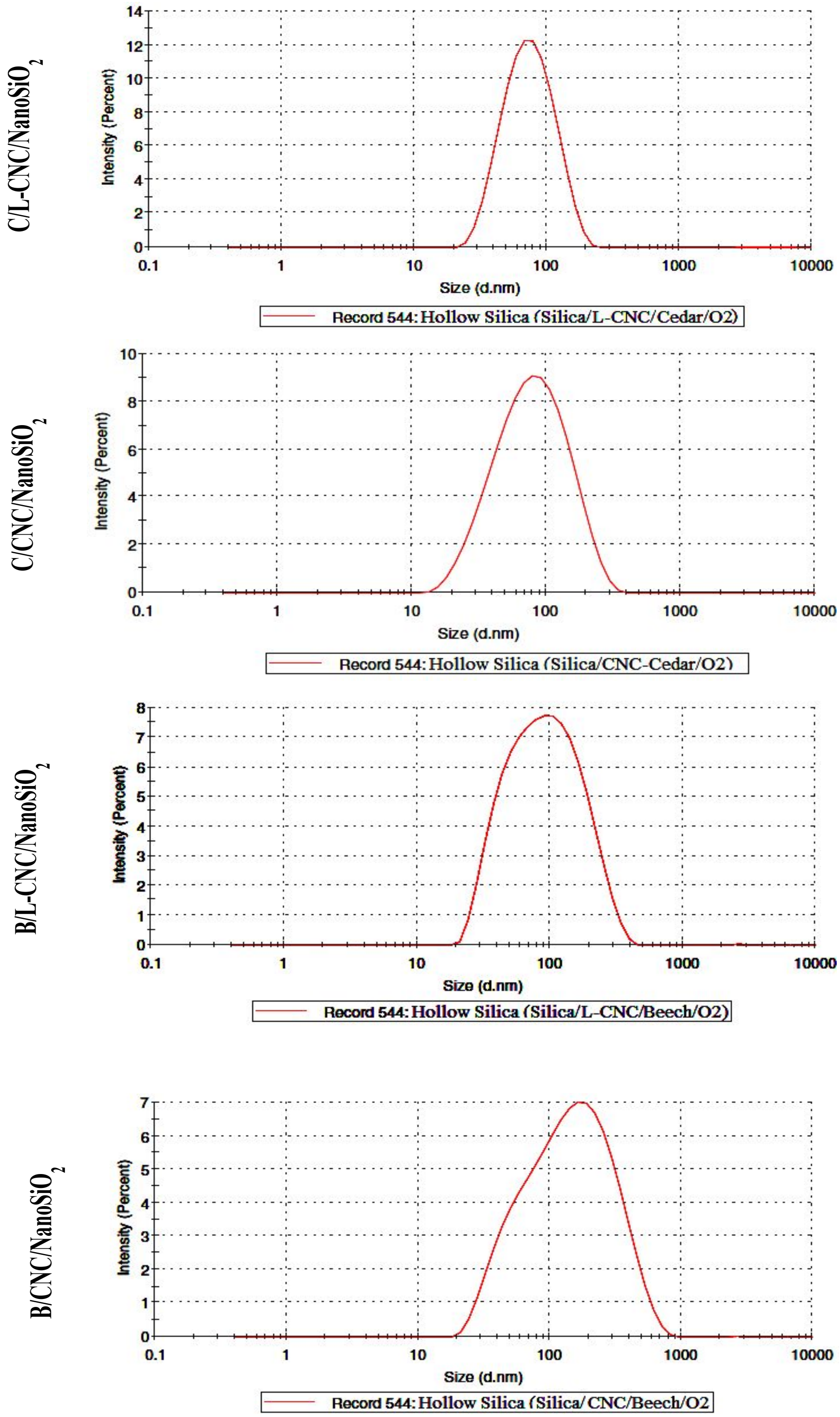

Figure S9: Particle size distribution of nanostructured porous silica materials

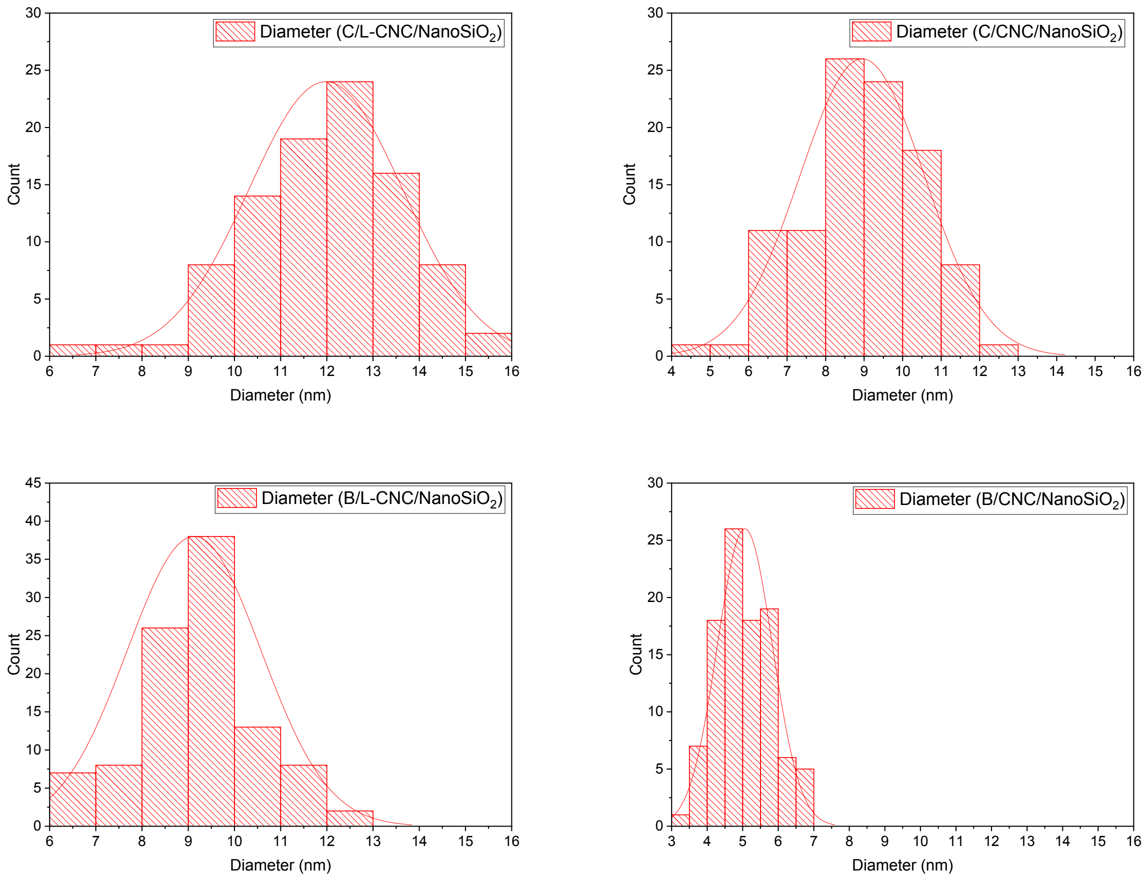

Figure S10: Inner diameter distribution of different hollow nano-silica

- **Determination of specific area by BET analysis**

Figure S11 presents the nitrogen adsorption-desorption isotherms curves of the different nanostructured hollow silica prepared by the intermediate of L-CNCs and CNCs cores from Cedar and Beech sawdusts.

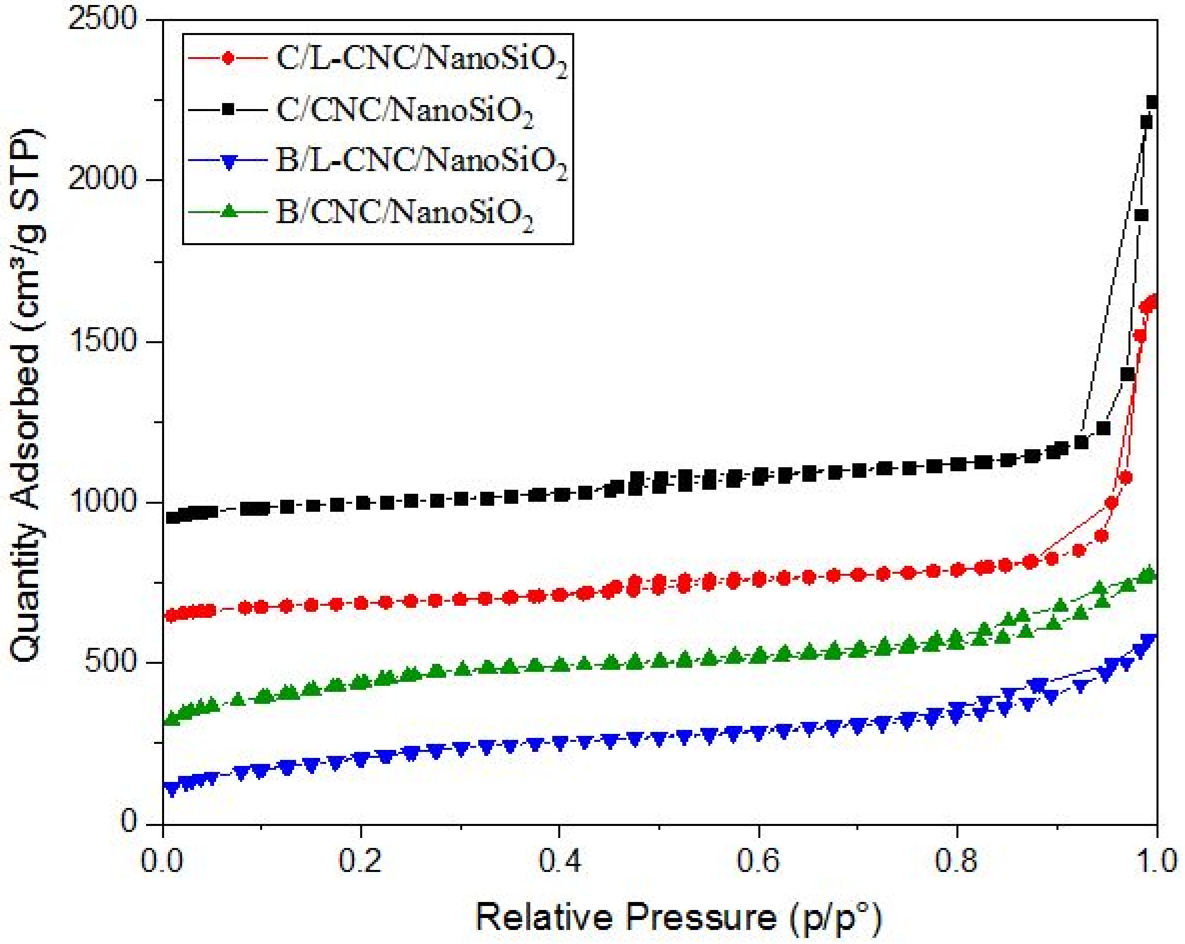

Figure S11: N_2 adsorption–desorption isotherms of nanostructured porous silica materials (each isotherm is shifted vertically for clarity)

References

[1] M. El Hajam, N. Idrissi Kandri, A. Harrach, A. El khomsi, A. Zerouale, Physicochemical Characterization of Softwood Waste "Cedar" and Hardwood Waste "Mahogany": Comparative Study, Mater. Today Proc. 13 (2019) 803–811. https://doi.org/10.1016/j.matpr.2019.04.043.

[2] E. Abraham, B. Deepa, L.A. Pothen, J. Cintil, S. Thomas, M.J. John, R. Anandjiwala, S.S. Narine, Environmental Friendly Method for the Extraction of Coir Fibre and Isolation of Nanofibre, Carbohydr. Polym. 92 (2013) 1477–1483. https://doi.org/10.1016/j.carbpol.2012.10.056.

[3] N.H. Abdul Rahman, B.W. Chieng, N.A. Ibrahim, N. Abdul Rahman, Extraction and Characterization of Cellulose Nanocrystals from Tea Leaf Waste Fibers, Polymers (Basel). 9 (2017) 1–11. https://doi.org/10.3390/polym9110588.

[4] M. El Achaby, N. El Miri, A. Aboulkas, M. Zahouily, E. Bilal, A. Barakat, A. Solhy, Processing and Properties of Eco-Friendly Bio-Nanocomposite Films Filled with Cellulose Nanocrystals from Sugarcane Bagasse, Int. J. Biol. Macromol. 96 (2017) 340–352. https://doi.org/10.1016/j.ijbiomac.2016.12.040.

[5] C.J. Chirayil, J. Joy, L. Mathew, M. Mozetic, J. Koetz, S. Thomas, Isolation and Characterization of Cellulose Nanofibrils from Helicteres Isora Plant, Ind. Crops Prod. 59 (2014) 27–34. https://doi.org/10.1016/j.indcrop.2014.04.020.

[6] V. Mikulcová, R. Bordes, A. Minařík, V. Kašpárková, Pickering Oil-In-Water Emulsions Stabilized by Carboxylated Cellulose Nanocrystals – Effect of the pH, Food Hydrocoll. 80 (2018) 60–67. https://doi.org/10.1016/j.foodhyd.2018.01.034.

[7] A.A. Oun, J.W. Rhim, Isolation of Cellulose Nanocrystals from Grain Straws and their Use for the Preparation of Carboxymethyl Cellulose-Based Nanocomposite Films, Carbohydr. Polym. 150 (2016) 187–200. https://doi.org/10.1016/j.carbpol.2016.05.020.

[8] M. Ristolainen, R. Alén, P. Malkavaara, J. Pere, Reflectance FTIR Microspectroscopy for Studying Effect of Xylan Removal on Unbleached and Bleached Birch Kraft Pulps, Holzforschung. 56 (2002) 513–521. https://doi.org/https://doi.org/10.1515/HF.2002.079.

[9] M. El Achaby, N. El Miri, H. Hannache, S. Gmouh, H. Ben youcef, A. Aboulkas, Production of Cellulose Nanocrystals from Vine Shoots and their Use for the Development

of Nanocomposite Materials, Int. J. Biol. Macromol. 117 (2018) 592–600. https://doi.org/10.1016/j.ijbiomac.2018.05.201.

[10] H. Zhang, Y. Chen, S. Wang, L. Ma, Y. Yu, H. Dai, Y. Zhang, Extraction and Comparison of Cellulose Nanocrystals from Lemon (Citrus Limon) Seeds using Sulfuric Acid Hydrolysis and Oxidation Methods, Carbohydr. Polym. 238 (2020) 116180. https://doi.org/10.1016/j.carbpol.2020.116180.

[11] H. Dai, S. Ou, Y. Huang, H. Huang, Utilization of Pineapple Peel for Production of Nanocellulose and Film Application, Cellulose. 25 (2018) 1743–1756. https://doi.org/10.1007/s10570-018-1671-0.

[12] K. Obi Reddy, B.R. Guduri, A. Varada Rajulu, Structural Characterization and Tensile Properties of Borassus Fruit Fibers, J. Appl. Polym. Sci. 114 (2009) 603–611. https://doi.org/10.1002/app.

[13] M. Sain, S. Panthapulakkal, Bioprocess Preparation of Wheat Straw Fibers and Their Characterization, Ind. Crops Prod. 23 (2006) 1–8. https://doi.org/10.1016/j.indcrop.2005.01.006.

[14] X.F. Sun, F. Xu, R.C. Sun, P. Fowler, M.S. Baird, Characteristics of Degraded Cellulose Obtained from Steam-Exploded Wheat Straw, Carbohydr. Res. 340 (2005) 97–106. https://doi.org/10.1016/j.carres.2004.10.022.

[15] K.Y. Goh, Y.C. Ching, C.H. Chuah, L.C. Abdullah, N.S. Liou, Individualization of Microfibrillated Celluloses from Oil Palm Empty Fruit Bunch: Comparative Studies between Acid Hydrolysis and Ammonium Persulfate Oxidation, Cellulose. 23 (2016) 379–390. https://doi.org/10.1007/s10570-015-0812-y.

[16] A. Alemdar, M. Sain, Isolation and Characterization of Nanofibers from Agricultural Residues - Wheat Straw and Soy Hulls, Bioresour. Technol. 99 (2008) 1664–1671. https://doi.org/10.1016/j.biortech.2007.04.029.

[17] C. Du, H. Li, B. Li, M. Liu, H. Zhan, Characteristics and Properties of Cellulose Nanofibers Prepared by TEMPO Oxidation of Corn Husk, BioResources. 11 (2016) 5276–5284. https://doi.org/10.15376/biores.11.2.5276-5284.

[18] A.M. Adel, Z.H. Abd El-Wahab, A.A. Ibrahim, M.T. Al-Shemy, Characterization of Microcrystalline Cellulose Prepared from Lignocellulosic Materials. Part II: Physicochemical Properties, Carbohydr. Polym. 83 (2011) 676–687.

https://doi.org/10.1016/j.carbpol.2010.08.039.

[19] H. Dai, Y. Huang, H. Huang, Eco-Friendly Polyvinyl Alcohol/Carboxymethyl Cellulose Hydrogels Reinforced with Graphene Oxide and Bentonite For Enhanced Adsorption of Methylene Blue, Carbohydr. Polym. 185 (2018) 1–11. https://doi.org/10.1016/j.carbpol.2017.12.073.

[20] H. Yu, Z. Qin, B. Liang, N. Liu, Z. Zhou, L. Chen, Facile Extraction of Thermally Stable Cellulose Nanocrystals with A High Yield Of 93% through Hydrochloric Acid Hydrolysis Under Hydrothermal Conditions, J. Mater. Chem. A. 1 (2013) 3938–3944. https://doi.org/10.1039/c3ta01150j.

[21] Y.W. Chen, H.V. Lee, J.C. Juan, S.M. Phang, Production of New Cellulose Nanomaterial from Red Algae Marine Biomass Gelidium Elegans, Carbohydr. Polym. 151 (2016) 1210–1219. https://doi.org/10.1016/j.carbpol.2016.06.083.

[22] M. Le Troedec, D. Sedan, C. Peyratout, J.P. Bonnet, A. Smith, R. Guinebretiere, V. Gloaguen, P. Krausz, Influence of Various Chemical Treatments on the Composition and Structure of Hemp Fibres, Compos. Part A Appl. Sci. Manuf. 39 (2008) 514–522. https://doi.org/10.1016/j.compositesa.2007.12.001.

[23] G. Müller, C. Schöpper, H. Vos, A. Kharazipour, A. Polle, FTIR-ATR Spectroscopic Analyses of Changes in Wood Properties During Particleand Fibreboard Production of Hardand Softwood Trees, BioResources. 4 (2009) 49–71. https://doi.org/10.15376/biores.4.1.49-71.

[24] S. Maiti, J. Jayaramudu, K. Das, S.M. Reddy, R. Sadiku, S.S. Ray, D. Liu, Preparation and Characterization of Nano-Cellulose with New Shape From Different Precursor, Carbohydr. Polym. 98 (2013) 562–567. https://doi.org/10.1016/j.carbpol.2013.06.029.

[25] K. Saelee, N. Yingkamhaeng, T. Nimchua, P. Sukyai, An Environmentally Friendly Xylanase-Assisted Pretreatment for Cellulose Nanofibrils Isolation from Sugarcane Bagasse by High-Pressure Homogenization, Ind. Crops Prod. 82 (2016) 149–160. https://doi.org/10.1016/j.indcrop.2015.11.064.

[26] H. Yang, R. Yan, H. Chen, D.H. Lee, C. Zheng, Characteristics of Hemicellulose, Cellulose and Lignin Pyrolysis, Fuel. 86 (2007) 1781–1788. https://doi.org/10.1016/j.fuel.2006.12.013.

[27] F. Kallel, F. Bettaieb, R. Khiari, A. García, J. Bras, S.E. Chaabouni, Isolation and

Structural Characterization of Cellulose Nanocrystals Extracted from Garlic Straw Residues, Ind. Crops Prod. 87 (2016) 287–296. https://doi.org/10.1016/j.indcrop.2016.04.060.

[28] S. Thambiraj, D. Ravi Shankaran, Preparation and Physicochemical Characterization of Cellulose Nanocrystals from Industrial Waste Cotton, Appl. Surf. Sci. 412 (2017) 405–416. https://doi.org/10.1016/j.apsusc.2017.03.272.

[29] Y.H.P. Zhang, L.R. Lynd, Toward an Aggregated Understanding of Enzymatic Hydrolysis of Cellulose: Noncomplexed Cellulase Systems, Biotechnol. Bioeng. 88 (2004) 797–824. https://doi.org/10.1002/bit.20282.

[30] J.W. Rhim, J.P. Reddy, X. Luo, Isolation of Cellulose Nanocrystals from Onion Skin and their Utilization for the Preparation of Agar-Based Bio-Nanocomposites Films, Cellulose. 22 (2015) 407–420. https://doi.org/10.1007/s10570-014-0517-7.

[31] H.Y. Yu, D.Z. Zhang, F.F. Lu, J. Yao, New Approach for Single-Step Extraction of Carboxylated Cellulose Nanocrystals for their Use as Adsorbents and Flocculants, ACS Sustain. Chem. Eng. 4 (2016) 2632–2643. https://doi.org/10.1021/acssuschemeng.6b00126.

[32] K. Nakasone, T. Kobayashi, Effect of Pre-Treatment of Sugarcane Bagasse on the Cellulose Solution and Application for the Cellulose Hydrogel Films, Polym. Adv. Technol. 27 (2016) 973–980. https://doi.org/10.1002/pat.3757.

[33] E. de M. Teixeira, T.J. Bondancia, K.B.R. Teodoro, A.C. Corrêa, J.M. Marconcini, L.H.C. Mattoso, Sugarcane Bagasse Whiskers: Extraction and Characterizations, Ind. Crops Prod. 33 (2011) 63–66. https://doi.org/10.1016/j.indcrop.2010.08.009.

[34] C.C.S. Coelho, M. Michelin, M.A. Cerqueira, C. Gonçalves, R. V. Tonon, L.M. Pastrana, O. Freitas-Silva, A.A. Vicente, L.M.C. Cabral, J.A. Teixeira, Cellulose Nanocrystals from Grape Pomace: Production, Properties and Cytotoxicity Assessment, Carbohydr. Polym. 192 (2018) 327–336. https://doi.org/10.1016/j.carbpol.2018.03.023.

[35] C. Jia, H. Bian, T. Gao, F. Jiang, I.M. Kierzewski, Y. Wang, Y. Yao, L. Chen, Z. Shao, J.Y. Zhu, L. Hu, Thermally Stable Cellulose Nanocrystals Toward High-Performance 2D and 3D Nanostructures, ACS Appl. Mater. Interfaces. 9 (2017) 28922–28929. https://doi.org/10.1021/acsami.7b08760.

[36] S. Andersson, R. Serimaa, T. Paakkari, P. Saranpää, E. Pesonen, Crystallinity of Wood and

the Size of Cellulose Crystallites in Norway Spruce (Picea Abies), Japan Wood Res. Soc. 49 (2003) 531–537. https://doi.org/10.1007/s10086-003-0518-x.

[37] A.D. French, Idealized Powder Diffraction Patterns for Cellulose Polymorphs, Cellulose. 21 (2014) 885–896. https://doi.org/10.1007/s10570-013-0030-4.

[38] A. El Oudiani, Y. Chaabouni, S. Msahli, F. Sakli, Crystal Transition from Cellulose I to Cellulose II in NaoH Treated Agave Americana L. Fibre, Carbohydr. Polym. 86 (2011) 1221–1229. https://doi.org/10.1016/j.carbpol.2011.06.037.

[39] S. Yildiz, E. Gümüşkaya, The Effects of Thermal Modification on Crystalline Structure of Cellulose in Soft and Hardwood, Build. Environ. 42 (2007) 62–67. https://doi.org/10.1016/j.buildenv.2005.07.009.

[40] R. Moriana, F. Vilaplana, M. Ek, Cellulose Nanocrystals from Forest Residues as Reinforcing Agents for Composites: A Study from Macro- to Nano-Dimensions, Carbohydr. Polym. 139 (2016) 139–149. https://doi.org/10.1016/j.carbpol.2015.12.020.

[41] Y.Y. Liu, J.L. Xu, Y. Zhang, C.Y. Liang, M.C. He, Z.H. Yuan, J. Xie, Reinforced Alkali-Pretreatment for Enhancing Enzymatic Hydrolysis of Sugarcane Bagasse, Fuel Process. Technol. 143 (2016) 1–6. https://doi.org/10.1016/j.fuproc.2015.11.004.

[42] A. Kumar, Y. Singh Negi, V. Choudhary, N. Kant Bhardwaj, Characterization of Cellulose Nanocrystals Produced by Acid-Hydrolysis from Sugarcane Bagasse as Agro-Waste, J. Mater. Phys. Chem. 2 (2020) 1–8. https://doi.org/10.12691/jmpc-2-1-1.

[43] N.A. Rosli, I. Ahmad, I. Abdullah, Isolation and Characterization of Cellulose Nanocrystals from Agave Angustifolia Fibre, BioResources. 8 (2013) 1893–1908. https://doi.org/10.15376/biores.8.2.1893-1908.
